# Bicuspid Aortic Valve: An Update in Morphology, Genetics, Biomarker, Complications, Imaging Diagnosis and Treatment

**DOI:** 10.3389/fphys.2018.01921

**Published:** 2019-01-30

**Authors:** Tianshu Liu, Mingxing Xie, Qing Lv, Yuman Li, Lingyun Fang, Li Zhang, Wenhui Deng, Jing Wang

**Affiliations:** ^1^Department of Ultrasound, Union Hospital, Tongji Medical College, Huazhong University of Science and Technology, Wuhan, China; ^2^Hubei Province Key Laboratory of Molecular Imaging, Wuhan, China

**Keywords:** BAV, morphology, genetics, biomarker, aortopathy, valvulopathy

## Abstract

The bicuspid aortic valve, a kind of heart disease that comes from parents, has been paid attention around the world. Although most bicuspid aortic valve (BAV) patients will suffer from some complications including aortic stenosis, aortic regurgitation, endocarditis, and heart dysfunction in the late stage of the disease, there is none symptom in the childhood, which restrains us to diagnose and treatment in the onset phase of BAV. Hemodynamic abnormalities induced by the malformations of the valves in BAV patients for a long time will cause BAV-associated aortopathy: including progress aortic dilation, aneurysm, dissection and rupture, cardiac cyst and even sudden death. At present, preventive surgical intervention is the only effective method used in this situation and the diameter of the aorta is the primary reference criterion for surgery. And the treatment effects are always not satisfactory for patients and clinicians. Therefore, we need more methods to evaluate the progression of BAV and the surgery value and the appropriate intervention time by combining basic research with clinical treatment. In this review, advances in morphology, genetic, biomarkers, diagnosis and treatments are summarized, which expects to provide an update about BAV. It is our supreme expectations to provide some evidences for BAV early screening and diagnosis, and in our opinion, personalized surgical strategy is the trend of future BAV treatment.

## Introduction

Bicuspid aortic valve (BAV) disease has the characteristic of heredity with variable genetic penetrance. And 0.5–2% of the population worldwide have the possibility to be attacked by this disease, which 75% of them are male (Dargis et al., 2016; Longobardo et al., 2016). Initially, most children with BAV disease are asymptomatic and not aware of its presence, resulting in complications which are presented in adulthood. Accurate diagnosis at early stage and effective intervention are essential to prevent various complications. Nature history of patients with surgery is not same as normal ones, therefore continued surveillance and personalized surgical therapy are required. Although a series of studies have introduced some aspects of BAV including etiology, rheology, morphology and genetics, questions are still unanswered (Masri et al., 2017b). This review provides a brief introduction of morphology characteristics, recent advances on genetic and biomarkers, and update in imaging diagnosis and recommendations for the treatment of BAV disease.

## Morphology: Different Classification Endow Different Effects

The typical structure of the aortic valve had three semilunar leaflets in shape. Due to the fusion of two cusps out of three, BAV usually included two unequal cusps and a central raphe.

**FIGURE 1:**
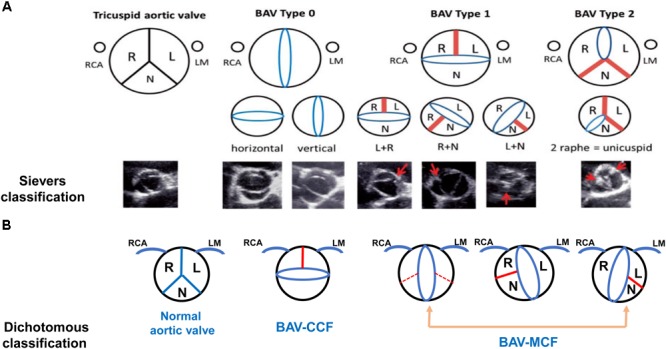
Schematic diagram and images of different bicuspid aortic valve morphology. The Sievers classification and echocardiographic images are shown in **(A)**. Dichotomous classification is shown in **(B)**. Red bands and arrows represent the raphe or commissural fusion. Red dotted lines represent that patients with BAV has the fusion of the non-coronary cusp with the right coronary cusp or the left coronary cusp but it is hard to demonstrate raphe to adequately classify the subtype. The ostium of right coronary artery (RCA) is depicted on the left and the left main (LM) is on the right (**A** used with permission).

From a surgical point of view, Sievers classification system was used widely in Figure 1A (Sievers and Schmidtke, 2007). Based on number of raphes, three categories of BAV are presented in patients including type 0 (no raphe in the valve), type 1 (only one raphe in the valve) and type 2 (two raphes in the valve). And the most common type is type 1, which accounting for about 90% of the patients (Sievers et al., 2014). On the basis of the raphe position with coronary sinuses, types 1 and 2 were classified as left (L), right (R) and none (N) type. The right and left coronary leaflet (RL) were most common accounted for about 80%, the right and non-coronary leaflet (RN) was about 17% and left and non-coronary leaflet (LN) was 2%. Compared to Asians, type 0 BAV was more frequently among Europeans, whereas the incidence of RN-BAV with a raphe was higher in Asian (Kong et al., 2018). Aortic stiffness was measured by pulse wave velocity (PWV) using velocity-encoded magnetic resonance imaging (VENC-MRI) and patients with R-NC fusion were manifested as greater PWV than patients with R-L fusion phenotype. This assessment might be a novel parameter to evaluate surgical risk (Boonyasirinant et al., 2018).

In order to show the relationship between BAV morphology type and valvulopathy or aortopathy, the dichotomous classification method was introduced in Figure 1B. The right and left coronary leaflet cusp fusion was defined as the coronary cusp fusion (CCF) and all other types were defined as the mixed cusp fusion (MCF). The MCF type of BAV was considered as one of risk factors for the occurrence of aortic stenosis and associated aortopathy, which resulted in significant hemodynamic changes. Transthoracic echocardiography (TTE) imaging that has characteristics of accuracy and feasibility makes this classification be a valuable approach in routine use (Jilaihawi et al., 2016; Sun et al., 2017).

Transthoracic echocardiography was the preferred method for the diagnosis of valvular status in BAV patients. The classical shape, “fish-mouth,” was appeared in the views of short-axis when the valve was step into systole. And the view of long-axis might also provide some abnormal manifestations of bicuspid valve in systole (Lee et al., 2015). TTE alone, however, is hard to determine presence/absence of raphe or position of the orifice of the coronary artery. TTE could identify valvular phenotype in 47.4% patients and 2D TEE might achieved to 90.1%. But, 2D TEE images was hard to provide the precise phenotype in 9.9% BAV patients, where 3D TEE was effective. In patients who had short raphe or disappearance of the raphe due to calcification in aortic leaflets, 3D TEE might excel in providing important features about valve structure in experienced hands (Yakar et al., 2017). And another imaging method, color doppler, is a useful tool to identify immobile trileaflet aortic valves or bicuspid valve by commissural fusion (Lee et al., 2015).

In order to preferable evaluate the aortic valve, the anatomy of aortic root especially the three-dimensional anatomy must be comprehended intensively. The aortic root is consisted of three distinct entities, including the sino-tubular junction (STJ), aortic sinus and the annulus. The aortic sinus contains the leaflets of aortic valve, the attachments of the leaflet, the sinuses of Valsalva and inter leaflet trigones. The annulus encompasses basal ring, the surgical annulus and the anatomical ventriculo-aortic junction (VAJ). The basal ring, an elliptical shape ring, is defined as a ring with three anchors at the bottom of each attachments of aortic cusps and calcification makes its shape non-homogenous. The surgical annulus, straddling the entire aortic root lengthwise from basal ring to STJ, is characterized as a complex three-dimensional crown-shaped structure, which the lowest parts are nadirs of the sinuses of Valsalva, and the highest ones are the commissures (Komiya, 2015). The ventriculo-aortic junction (VAJ) is a circle which is positioned slightly above the basal ring between the aortic wall and left ventricular myocardium. Patients who received the aortic valve repair, the mean diameter of VAJ in tricuspid exceeding 26 mm and the mean diameter of VAJ in bicuspid valve exceeding 28 mm are defined as relative VAJ enlargement (de Kerchove et al., 2012).

The functional aortic annulus (FAA) which seems as elliptical was surrounded by VAJ and STJ at the aortic root. In order to interpretation the different dilatation in all FAA, El et al. (2005) recommended the classification of FAA which aimed to recognize different type of aortic regurgitation. In clinical, the most common phenotypes in BAV repair are El Khoury types Ib and II, which based on Sievers type 1 L-R BAV (Bavaria et al., 2013). Type-IB AR is caused by sinuses of Valsalva dilatation which is excised and replaced with the help of reimplantation or the remodeling technique. Type-II AR is secondary to excessive cusp or commissural disruption, which is corrected with interrupted sub-commissural annuloplasty plication sutures (El et al., 2005). It is suggested that three dimensions transesophageal echocardiography (3D TEE) and multidetector computed axial tomography (MDCT) were effective in evaluating FAA and most important of all, MDCT is recommend as a criterion imaging technique used to device sizing in the operation of TAVR (Jilaihawi et al., 2012). CT area possessed the highest correlation and the best agreement with intraoperative sizing, which leading 84.6 and 74.0% of BAVs to agree for theoretical surgical with TAVR prosthesis selection (Patel et al., 2015). Through 3DTEE could be used as an alternative for CT in BAVs patients who unsuitable for CT, it still need to be paid more attention especially in patients with aortic annulus calcification because inaccuracy could be caused by partial acoustic shadowing (Wang Y. et al., 2018).

In the era of TAVR, it is important to understand the aortic root anatomy precisely, which is the basis for device development, selection and implantation. In BAV patients, the sizes of annulus, sinus of Valsalva and ascending aorta have been enlarged. Traditionally, exclusion diagnosis is the standard diagnostic method for BAV because of asymmetric aortic annulus. This anatomical feature might cause leaflet malformation, even para-valvular leak due to asymmetric deformation and expansion of the rounded bioprosthesis (Zegdi et al., 2008). However, the larger annulus of BAV seems to be a circle and could be suitable for current commercial valved stents (Philip et al., 2015).

Sizing the aortic valve annulus that is representing the tightest section of aortic root based on the anatomy, which is suggested to be a valuable method in selecting the size of transcatheter heart valve (THV). The morphological characteristics of supra-annulus that is a scope locating from annulus to the STJ is becoming significantly complex when bicuspid AS combines with severe calcification in leaflets. Annulus measurements using CT might be inaccurate because the definition of the annulus plane derive from two hinge points of the leaflet. The supra-annular structure might have great effects on anchoring THV. Patients who have been diagnosed BAV manifest as morphological changes in valve including annular eccentricity, asymmetrical calcified leaflet, presenting different sizes of leaflets, and following by aortopathy, which increases the possibilities of deeper implantation, PVL, TAV-in-TAV, annulus rupture, aortic dissection (Zhao et al., 2015). Though CT have advantages in providing anatomic aortic root information, the mechanical characteristics of the annulus or supra-annular structure for THV anchoring could not be revealed completely. However, Balloon sizing, not descripted before, could provide the information about supra-annular mechanical characteristics through the sign of balloon waist combination with contrast aortogram and AR evaluation (Liu et al., 2018).

It is critical to select appropriate valve size after TAVI, which could promote rehabilitation. As a valid compensation for multimodality imaging, Balloon sizing has its own characteristics. Although there is smaller anticipated sizing ratio, the incidence of PAR after valve deployment and short-term follow-up does not increase after switching to smaller THV with balloon sizing. With the help of MSCT, patients with smaller valves possessed better stent frame expansion at inflow and a trend toward better frame geometry in terms of circularity than those who received valves of the same size or larger. Although MSCT has extraordinary effect in THV size selecting, balloon sizing could be used as a complementary tool as well as for BAV morphology assessment (Xu et al., 2018).

Early repair failure is associated with failure to address the functional aortic annulus in the process of BAV repairment. The deep anterior dissection technique not only avert the problem, but also acquires another goals including making the annuloplasty ring surrounded by the basal ring, permitting to symmetrical implantation, making the tissues of the root anteriorly become thin, causing less muscle bands and improving the accuracy of grafting sizing and matching and preventing recurrence of dilatation in the same area and prolapse of conjoined cusp by IVS (Nawaytou et al., 2018).

However, we speculated that aortic valve annulus eccentricity might be innate in infant and early child period. Children who manifest as severe or critical aortic stenosis usually are treated with aortic balloon valvuloplasty, rather than transcatheter AV replacements. 2D angiographic examination of parasternal long axis could assess the size of the aortic valve annulus in pediatric patients at present. The incidence of aortic insufficiency is increasing, as well as residual stenosis, when the size of eccentric annulus is inaccuracy. We suggest that aortic ballon valvuloplasty might help with therapeutic effects when taking the different annular size into account (Chamberland et al., 2015).

## Genetics: Polymorphism and Complexity of the Pathogenesis

It is well known that the genetic etiologies of BAV have the characteristic of diversity, which has the same characteristic with BAV phenotypes. BAV was an autosomal-dominant disease and had a highly heritable trait which was up to 89% (Freeze et al., 2016). Several investigators had shed light upon the mechanism induced by gene mutation in the development of BAV. NOTCH1 mutations were highly penetrant (75%), most of which were BAV (Kerstjens-Frederikse et al., 2016). NOTCH1 combined with different ligands at different stages of morphogenetic processes to express variant regulatory effects. Delta-like 4 (Dll4), one of NOTCH ligand, was necessary for epithelial-to-mesenchyme transition formation, and endocardial Jag1-NOTCH1 signaling was essential for later morphogenesis and regulation of mesenchyme proliferation in post epithelial-to-mesenchyme transition (MacGrogan et al., 2016). Additionally, NOTCH1 could affect calcium deposition through inhibition Runx2 activation. In the shear stress condition, NOTCH1 haploinsufficiency led to calcification of endocardial cells by dysregulating the downstream transcription of genes, inflammation, oxidative stress and aberrant upregulating pro-calcific nodes (Theodoris et al., 2015). It has been reported that endothelial progenitor cells (EPC) played a critical role in vascular repair processes during stress and development of aortic aneurysms. Compared with TAV patients, the expression level of Notch gene in tissue and circulation was significantly reduced in BAV patients, which directly reduced circulating EPC number, either in presence or absence of ascending aortic aneurysm (Balistreri et al., 2018).

The GATA family is a class of zinc finger proteins. The sub-group, GATA5, was a transcription factor, which was expressed in an overlapping together with GATA4 and GATA6 (Padang et al., 2012). The fusion of aortic valve in GATA5 deletion mice was presented in posterior intercalated cushion and septal ridge, resulting in RN BAV. Hence, the effect of GATA5 mutation might be more important in RN type BAV patients (Laforest et al., 2011). GATA4 was critical for heart differentiation. Two genetic variants have been identified that approached even achieved to genome-wide levels. The rs6601627, representing a low-frequency intergenic variant, has a higher incidence rate in BAV patients. The rs3729856 variant was associated with protein expression, which involved in specific gene transcription in cardiac (Yang et al., 2017). GATA6, another GATA family member, might be a novel BAV causing gene. GATA6 heterozygous mice had highly penetrant RL type BAV, the most common type in human. GATA6 haploinsufficiency undermined valve remodeling and extracellular matrix composition through dysregulation of important signaling molecules including matrix metalloproteinase 9. Defective valve remodeling due to dysregulated extracellular matrix degradation has been suggested to be potential mechanism in BAV formation (Michelena et al., 2014). But common variants at the GATA6 locus associated with BAV were unclear. Of note, although the relation between GATA6 and BAV in human has not been illuminated, the prevalence of BAV is greater in male GATA6+/- mice than female (Gharibeh et al., 2018).

Other mutated gene often correlated with specific features and prognosis. Due to abnormal flow in BAV aneurysm, the aberrant methylation of epithelial-mesenchymal-transition and increased expression of WNT/β-catenin genes like MYLK, ZEB1 were involved (Bjorck et al., 2018). Besides, transcriptome analyses and immunohistochemical study of ascending aortic specimens from BAV patients showed that receptor tyrosine kinase (RTK)/p-AKT pathway in the middle part of ascending aortic wall was activated, in spite of the constant expression level of total AKT (Hirata et al., 2018; Yamashita et al., 2018). Mutations in ACTA2, FBN1, and TGFBR2 genes might be the reason of BAVs with thoracic aortic aneurysm (Giusti et al., 2017). Two new aortic valve stenosis loci, named rs7543130 on chromosome 1p21 near PALMD or rs1830321 on chromosome 2q22 in TEX41, might be involved in the development of BAV. Rs7543130 associated with BAV and aortic root diameter, and rs1830321 associated with BAV and coronary artery disease (Helgadottir et al., 2018). Genetic mutations above determining bicuspid aortic valve are summarized in Table 1.

**Table 1:** Main genetic mutations determining bicuspid aortic valve.

Gene	Mechanism	Reference
NOTCH 1	Affect epithelial-to-mesenchyme transition formation and calcium deposition and reduce circulating EPC number	Theodoris et al., 2015;Kerstjens-Frederikse et al., 2016;MacGrogan et al., 2016;Balistreri et al., 2018
GATA 5	Associated with RN BAV formation	Laforest et al., 2011
GATA 4	Impair endothelial-to-mesenchymal transition	Yang et al., 2017
GATA 6	Undermine valve remodeling and EMC by dysregulation of MMP9	Michelena et al., 2014
ACTA2, FBN1, TGFBR2	Involved in thoracic aortic aneurysm caused by BAV, Marfan syndrome, Loeys-Dietz syndrome	Giusti et al., 2017
WNT/β-catenin or RTK/p-AKT	Aberrant signaling pathway involved in BAV ascending aortic and aneurysm	Hirata et al., 2018;Yamashita et al., 2018
New loci on chromosome		
1p21 near rs7543130	Associated with increased aortic root size in BAV	Helgadottir et al., 2018
2q22 in rs1830321	Associated with BAV and coronary artery disease	

## Biomarkers of BAV: Prospective in Diagnosis and Treatment

Although BAV is a hereditary disease and most BAV patients are asymptomatic at onset, we still hope to explore some molecular biological markers to speculate the progression of BAV. Matrix metalloproteinase (MMP) would affect degradation of extracellular matrix proteins such as elastin and collagen in the aortic wall (Phillippi et al., 2009). The increased MMP-2 level in circulation could predict the ascending aortic dilation in BAV patients independently without significant valvular dysfunction (Wang et al., 2016; Wang Y.B. et al., 2018). TGF-β and its receptor ENG correlated with the severity of the development of aortic dilation in bicuspid aortic stenosis patients. The signaling pathway played a vital role in fibrosis, inflammation and extracellular matrix of cardiovascular system. There might be a relationship between TGF-β1/ENG ratio and aortic diameter. When aortic diameter <45 mm, the ratio becomes higher and abnormal aortic wall remodeling comes even more severer with increasing expression of MMP-2 and TGF-β as well as decreasing expression of superoxide dismutase 3. And, the ratio has positive correlation with the rate of ascending aortic growth in AVR postoperative period of bicuspid aortic stenosis patients. Therefore, the ratio of TGF-β1/ENG might be a novel predictor for the risk and severity of BAV aortopathy (Forte et al., 2017). However, the development of aneurysms in patients with BAV was not associated with an increased TGFβ activity (Paloschi et al., 2015). In stenotic aortic leaflets from BAV patients compared with TAVs, the downregulation of miR-195 was associated with accelerated valvular calcification via targeting SMAD7, which involved the progress of fibrosis and remodeling of the extracellular matrix (Du et al., 2017). In addition, miR-17 could indicate different part of aorta dilation by regulating expression of TIMP1/2 and MMP2. In normal aorta, the expression of miR-17 and related miRNAs was low. And the level of TIMP1/2 was normal, inhibiting MMP2 activity. During the early phase of BAV-associated dilation, miR-17 was upregulated, inhibiting TIMP1/2, which subsequently increased MMP-2 activity, leading to progressive extracellular matrix breakdown, aortic dilation and aneurysm formation. Once severe tissue dilation had occurred, miR-17 expression was reduced, TIMP1/2 expression increased, and MMP-2 activity decreased toward baseline levels (Wu et al., 2016). The expression of proteins and miRNAs might be a potential prognostic biomarker for predicting pathological aorta in BAV patients. Optimal therapies targeted at different proteins and miRNAs were not only novel treatments of aortic complications in BAV patients, but also preventing early stages of disease development before irreversible valve calcification, aorta became dilated severely and aortic aneurysm was formed aggressively. To date, the research about biomarkers that was used to early diagnose BAV clinically is still at its infancy. However, the truth on those markers has not been uncovered. And further investigations in large patient cohorts are needed to provide strong data.

## BAV Associated Complications: the Supplement for Guideline Diagnosis and Treatment

The BAV was not only just a valve disease, but also it was related to secondary aortic stenosis, aortic insufficiency, infective endocarditis and cardiac dysfunction. Due to the influence in gene and abnormal aortic blood flow, BAV patients would suffer from ascending aortic dilated and aneurysm. So far, the most strategic therapy is operation at appropriate time and prevent complications. In this part, we provide a brief introduction and supplement of diagnose and treatment for BAV.

## Bicuspid Valvulopathy: a Complication With High Incidence and Induced Left Ventricle Dysfunction Need to Pay More Attention

A recent report about prevalence and complications of BAV in Chinese, suggested that almost half of the patients who were diagnosed BAV had different degrees of aortic valve disease with similar results in western report (Li et al., 2017; Masri et al., 2017b). In the large international registry of 2118 patients with BAV, compared with patients with raphe, the prevalence of aortic stenosis and aortic regurgitation was lower in patients who have no raphe. The 2 raphes was associated with higher prevalence of significant aortic stenosis and regurgitation compared with the 1 raphe. Among patients with 1 raphe, RN-BAVs had more aortic stenosis. Due to valvular dysfunction at a younger age, BAV patients with raphe prefer to perform AVR, but not related with an increased risk for all-cause mortality (Kong et al., 2017).

## Aortic Stenosis: Multiple Imaging Technology Evaluation

As one of major risk factors, BAV was involved in mild to severe AS. TTE has been recommended in bicuspid aortic valve patients with symptoms. It could provide precise diagnosis of abnormal hemodynamic and left ventricle function. And, TTE might be used to decide the surgical intervention time and speculate prognosis (Nishimura et al., 2014). TTE derived left ventricular outflow tract (LVOT) area might be inaccurate because of single diameter of elliptical aortic root. Compared with the aortic valve area-ECHO (AVA ECHO), the AVA CT (aortic valve area was calculated by MDCT) was larger overall in BAVs, but could not show the relation to transvalvular gradient, the concordance gradient-AVA, or speculated mortality. When AVA was evaluated with CT (<1.2 cm) and Echo (<1.0 cm), larger cut-point values might be a valuable parameter in severe AS diagnosis (Clavel et al., 2015). Fusion imaging might provide a more accurate assessment of the “true” AVA by decreasing the rate of discordant AS with low gradient but increasing the rate of discordant AS with high gradient (Arangalage et al., 2018). In clinical, it is crucial to accurately diagnose the severity of AS with the help of routine doppler because of characteristics of multiple imaging windows (Thaden et al., 2015). Ejection dynamics, particularly acceleration time (AT) and the ratio of AT to ejection time (ET), could be applied to evaluate AS severity (Gamaza-Chulian et al., 2017). In addition, doppler ultrasonography was used to evaluate transvalvular pressure drop using the simplified Bernoulli formulation to measure the severity of AS. Recently, a formulation that is corrected to calculate the cross-sectional profile of blood flow was used to estimate peak pressure drops accurately (Donati et al., 2017). Cardiac computed tomography could be recommended in younger BAV patients could with higher sensitivity and specificity. Although valvular calcification of the BAV had a faster progress in younger patients, there might be a hemodynamically significant stenosis without aortic valve calcification (Shen et al., 2017). As WSS is important in evaluating viscous shear forces on vessel wall due to abnormal flowing blood. 4-dimensional (4D) flow MRI measured volumetric velocity vector fields which could represent the increased regional WSS (Dyverfeldt et al., 2015). Increased WSS in bicuspid AS patients distributed mainly along the ascending aorta, especially at the outer curvature. This was consistent with the fact that aortic dilatation in BAV patients was asymmetrically. The degree of elevated WSS was negatively related to aortic diameters. Therefore, in BAV patients, the elevated WSS of the ascending aorta was related to aortic valve stenosis and aortic dilatation, which was more obviously when patients manifested as AS and nondilated ascending aorta (Farag et al., 2018). If decomposed the WSS vector field into its axial and circumferential components (WSSA and WSSC) from 4D flow MRI data using a Laplacian approach, the average WSSC in the ascending aorta was statistically significant difference as a parameter for evaluating multidirectional flow in complex geometries (Sotelo et al., 2018).

In recent years, the proportion of bicuspid aortic stenosis patients who has been carried out TAVR is increasing with a tendency to younger patients. However, TAVR might meet different procedural changes. For Sievers type 0 patients with no raphe, the challenge might be an ellipse aortic annulus than a circle and the risk for paravalvular leakage. LR BAV patients might confront with the risk of imprecise allocation, due to the calcified raphe which placed unequal stress on the dilatation of a TAVR (Ben-Dor and Stewart, 2017). In respect of procedural threat of TAVR, the new generation devices including ballon-expandable Sapien 3 and Lotus valve could overcome the crucial limitation of previous devices such as obvious perivalvular leak, failure to get optimal positioning and associated vascular complications (Mylotte et al., 2014; Yang et al., 2015; Perlman et al., 2016; Yoon et al., 2016, 2017; Sannino et al., 2017). Transcatheter balloon aortic valvuloplasty was considered palliative therapy for BAV as well as a useful tool in sizing ascending aorta. Currently operators used a median effective final balloon to aortic valve annulus ratio of 0.94 as criterion (Boe et al., 2017). However, the optimal approach for sizing in bicuspid aortic stenosis was controversial. Some recommended perimeter/area at the annulus level as criterion, and the others suggested to use the commissural level above the annulus about 4–8 mm as the sizing selection (Ben-Dor and Stewart, 2017). To date, TAVR with the help of the new-generation device is a reasonable treatment option with the good outcome and low complication rate for bicuspid AS patients.

## Aortic Regurgitation: Different Surgical Interventions

Aortic regurgitation is more frequent in the earlier period of patient’s life than stenosis (Okita, 2015). Once aortic regurgitation is formed, the major factor affecting the development is left ventricular pathological change due to chronic volume overload. As a result, surgical intervention will be the final decision for most bicuspid AR patients (Braverman et al., 2005). We have a brief introduction including an assessment of degree of bicuspid AR and left ventricular function and different surgical interventions.

Transthoracic echocardiography is a preferred imaging test for evaluating bicuspid AR patients and guiding appropriate management decisions. Cardiac catheterization might be a valuable method to evaluate abnormal hemodynamics, anatomical structure of coronary artery and development of aortic regurgitation (Nishimura et al., 2014). Eccentric jet was common in BAV valve malformation, and it was difficult to accurately assess bicuspid AR with ultrasound. When eccentric jet was hard to be quantified and LV dilation was disproportionate to the degree of AR, cardiac MR might give a better way to quantity AR and LV volumes (Mojazi-Amiri and Pai, 2013). In a study on the character of speckle-tracking echocardiography in terms of judgment cardiac function, assessment of circumferential as well as longitudinal strains was important. For asymptomatic patients with hemodynamically significant bicuspid AR, global circumferential strain (GCS) and global longitudinal strain (GLS) were lower compared with healthy. GCS compensated for the reduced GLS to ensure that global function and left ventricular ejection fraction (LVEF) remained in the normal range. However, the study showed no association between LVEF and GLS, whereas GCS was correlated with LVEF (Broch et al., 2017).

The majority of patients would require AVR, exception of severe AR with LVEF ≥ 50%, LVESD ≤ 50 mm, LVEDD ≤ 65 mm or progressive AR without other cardiac surgery. The most frequent valve-related complication leading to re-operation had been recurrence of AR induced by dilatation of the aortic root. For mild bicuspid BAV patients with aortic root dilation (>40 mm) during their initial AVR procedure, the surgical intervention could be considered to decrease the incidence of recurrent AR (Girdauskas et al., 2017). If the regurgitation BAV patient combines with annular dilatation, the repair treatment might not be successful. Sub-commissural annuloplasty resulted in earlier repair failures compared with complete circumferential annuloplasty (Navarra et al., 2013; Nawaytou et al., 2018). Suture annuloplasty with polytetrafluorethylene as material could eliminated annular dilatation and reduce local complications and tissue trauma. Suture annuloplasty should be considered whenever basal diameter exceeds 26–27 mm. The stability could be comparable to valve preserving aortic root replacement without the associated complexity of the procedure. After aortic valve repair, the height of coaptation was considered acceptable if vena contracta was ≥4 mm on TEE (Schneider et al., 2017). Valve-sparing aortic root replacement (VSSR) could be a promising option for aortic regurgitation patients, which might improve repair durability, especially annulus stabilization, and should be recommended in young patients (Komiya, 2015).

## Infective Endocarditis: Antibiotic Prophylaxis Comes First

BAV patients were more likely to have infective endocarditis (IE) than the others (Siu and Silversides, 2010). The majority of BAV with IE were men. Adverse events of IE included heart failure, peripheral embolism, embolic stroke, persistent bacteremia, and intracardiac complications (Chambers, 2018; Zegri-Reiriz et al., 2018).

The most common organisms causing IE were microorganisms presented in the oral cavity, mainly viridians group streptococci. BAV should be considered as high-risk cardiac conditions with unrestricted IE antibiotic prophylaxis (Chambers, 2018). The signs of IE in TTE performance included vegetation, valvular perforation, and perivalvular abscess. There were no significant differences among left atrial diameter (LAD), left ventricular end-diastolic diameter (LVEDD), and left ventricular end-systolic diameter (LVESD) values like the incidences of serious AR or AS. Given the high frequency of perivalvular abscesses in BAV IE patients, systematic use of TTE or TEE in all BAV IE patients and prompt diagnosis of perivalvular abscesses are necessary and advantageous for the prognosis (Chen et al., 2017). In order to treat infection and avoid the sequelae resulting from tissue destruction of valve and paravalvular, surgical intervention was recommended, especially at early stage. To date, there was few long-term in multicentric follow-up study for BAV patients with infective endocarditis to verify the rationality of the current guidelines. For patients with BAV, cardiac echocardiography should be taken regularly to prevent the disease before getting worse.

## Left Ventricle Dysfunction: Important but Unclear Relationship With BAV

Under the presence of abnormal hemodynamics and aberrant gene expressions like TGF-β, NOTCH and MMPs, it might affect left ventricular function and lead to LV remodeling including myocardial hypertrophy and fibrosis. Although we did not have clear understanding about the relationship between BAV and myocardial hypertrophy, the data has showed that 0.9% of the HCM patients suffered from BAV and 0.4% of BAV patients were coexistent with HCM. 11 patients developed into dynamic obstruction, which always affect the LVOT. Resting obstruction was presented in five patients, which presented the gradient ± 30 mmHg (Padang et al., 2018). Collagen volume fraction (CVF) was reduced in sub endocardium and increased in endocardium. This might imply endocardial fibrosis in severe AS and AS was one of major complications of BAV as mentioned before. We speculate that endocardial fibrosis might exist in part of BAV patients (Treibel et al., 2018). Late gadolinium enhancement (LGE) on MRI imaging has been used to identify the presence, pattern, and size of fibrosis as well as myocardial and cardiomyopathy (Ambale-Venkatesh and Lima, 2015; Gaztanaga et al., 2016). In a retrospective study with 29 BAV patients, CMR and TTE images were reviewed. Patients with LGE had significantly higher aortic valve mean gradients by TTE and were more likely to have LV hypertrophy. They were more likely to need aortic valve replacement within 1 year. Evaluation of LGE by CMR as a marker of LV myocardial fibrosis could have additional prognostic value (Lluri et al., 2017). CMR is a non-invasive approach used to measure LV mass and volume and a valuable tool used to describe the LV remodeling in response to AS, which is regarded as a gold standard. BAV patients were demonstrated to have significantly larger left ventricular outflow tract dimensions (LVOTd), systolic LV area, LV end-diastolic volume (LVEDV), LV end-systolic volume (LVESV). The LVOTd is significant related with maximal aortic root diameters at all levels in BAV patients, especially with the aortic annulus diameter. But the correlation between LVOTd and mid-ascending aortic diameter was weak (Disha et al., 2017). The blood flow is strong eccentricity, which is presented in all degrees of AS. Alteration in poststenotic blood flow when AS is presented is the reason for elevating LV after load. The relationship between LV remodeling and aortic orifice area (AOA) emphasizes the notion that remodeling is a characteristic of all degrees of AS. The intensity of abnormal blood flow increases with the decreasing of AOA, and increases further with the presence of BAV. In addition to AOA, it is suggested that the flow parameter normalized flow displacement is an alternative sign for defective blood transporting and eccentric myocardial stress because it is in connection with LV remodeling. Different grades of AS were involved in elevated and asymmetrical WSS distribution. However, there is no correlation between it and LV remodeling. And the mechanotransduction risks of poststenotic dilatation should be paid more attention as it might happen in early period of AS (von Knobelsdorff-Brenkenhoff et al., 2016). The mass index and indexed volumes of LV or the ratio of LV mass/volume are always measured to explore the extent and patterns of hypertrophy. A specific wall thickening of the myocardial, no less than 13 mm or 1.5 times larger than the opposing one, is regarded as asymmetric remodeling and hypertrophy. There are as many as six patterns of LV adaption including normal ventricular, concentric remodeling, asymmetric remodeling, concentric hypertrophy, asymmetric hypertrophy and LV decompensation. Despite unstable, the increased stress of LV still could cause extensive LV impairment (Dweck et al., 2012). TTE is used to assess the systolic longitudinal strain which is a valuable indication to early LV disorder of AS. Decreasing in LV ejection fraction indicates that the LV dysfunction is stepping into the late stage (Ng et al., 2011; Pibarot and Dumesnil, 2012). However, there seems to be no significant correlation between pattern of LV adaption or degree of hypertrophy and BAV classification. Although the women BAV patients were more likely to have aortic stenosis, there was no significant differences in sex regarding left ventricular function assessed by cardiac MRI (Dweck et al., 2012).

In some BAV patients without aortic valve dysfunction and ascending aortic dilatation, cardiovascular MR tissue tracking imaging has demonstrated that ascending aortic and LV systolic and diastolic myocardial mechanics in all the three vectors (longitudinal, circumferential, and radial) are significantly impaired. The aortic valve congenital abnormality seems to be the only reason to cause LV impairment. That is to say, BAV may be not only a disease of aortic valve and aortic wall but also an inherent disease of the LV myocardium. This is a compelling evidence that the remodeling process of LV is associated with BAV, which has been debated for long (Nucifora et al., 2018). For asymptomatic BAV patients with preserved EF, feature-tracking CMR (CMR-FT) may be a useful and practical tool for assessing LV function. The impaired diastolic function caused by myocardial hypertrophy and fibrosis precedes systolic abnormalities and could predict the outcomes of surgical in patients with aortic stenosis. In asymptomatic BAV patients who have no signs of ventricular dysfunction by routine cardiac MRI, CMR-FT strain analysis can detect myocardial dysfunction especially when the diastolic strain rate decreased. With the improvement of surgery and diagnosis which could identify patients at younger age, the survival time of post-surgery patients has been extended. But continues monitoring LV function is still necessary. More attention should be paid to changes about cardiac structure and function to prevent LV dysfunction.

## BAV-Associated Aortopathy: More Criterion About Assessment and Treatment Should Be Included Expect for Aortic Diameter

The BAV-associated aortopathy has a prevalence of 40% in patients of multiple clinical centers. In contrast to general population, BAV patients were 86 times to develop proximal aortic aneurysms and 8 times to have aortic dissection. The morbidity of aortic dissection and rupture were about 0.4% during 2–16 years. The proportion of aortic rupture was very low, which there is only one rupture in all 14 cases in a 32 months follow-up (Michelena et al., 2011; Kim et al., 2016; Masri et al., 2016). Cardiac cyst originated from bicuspid aortic valve was an extremely rare type of cardiac mass (Gu et al., 2014).

## Aortic Dilatation: the Onset of BAV-Associated Aortopathy

Currently, there are two main theories on the relation between BAV and aortic dilatation: an underlying genetic substrate and hemodynamics theory. Gene mutations as mentioned above could not only causing the development of BAV but also exacerbating calcium deposition on the valve. The activity of MMPs is increased and fibrillin-1 expression is decreased in the wall of ascending aorta (Nataatmadja et al., 2003). Gradually, decreased collagen, increased elastic fragmentation, and reduced VSMCs in aortic middle layer degeneration lead to aortic convex surface before ascending aortic dilatation. And abnormal hemodynamics inside the ascending aortic will also cause uneven shear stress on the same area (Cotrufo et al., 2005). This has a promotional effect in increasing expression of MMPs and VSMC apoptosis (Lehoux and Tedgui, 2003). And a stronger hemodynamic influence might play an important role in BAV patients who have tubular and stiff ascending aortic dilation (Yassine et al., 2017). Not the stiffness index of all the aortic segments was higher than healthy. After adjusting the age and the aortic diameter, the sinus of Valsalva appeared to be the only segment significantly stiffer, which might suggest a local remodeling process in BAV patients (Goudot et al., 2018).

Aortic dilatation with BAV was classified as three types: type 1 was dilatation of root, ascending and proximal arch aorta dilation along convexity of aorta. Type 2 was relative normal root with tubular ascending aorta dilation. Type 3 was isolated dilatation of aortic root alone (Verma and Siu, 2014). The location of aorta dilation was related to valve morphology, sex and bicuspid valvulopathy. RN-BAVs and RL-BAVs would induce dilation of different aortic part, which the dilation induced by RN-BAVs was located in ascending aorta, whereas the one induced by RL-BAVs was aortic root (Ruzmetov et al., 2015; Krieger and Hung, 2018). AR could result in diffuse enlargement including annular, sinus of Valsalva, and ascending aortic, whereas AS of moderate to severe grade only led to ascending aortic enlargement. The diameter of sinus of Valsalva was larger in men when compared with women, whereas ascending aortic diameters were comparable (Roman et al., 2017). In addition, abnormal ratio of ascending aortic area/height, as well as sinus of Valsalva and ascending aortic dilatation rate might be regarded as predictors of cardiovascular death (Detaint et al., 2014; Masri et al., 2017a).

2D TTE is a feasible, accurate, and reproducible method for the non-invasive assessment of thoracic aortic diameters. It has been reported that unbiased agreement could be obtained between the CTA inner edge–to–inner edge method and TTE leading edge–to–leading edge method for the sinuses and ascending aortic (Park et al., 2017). Aortic WSS correlated with regional elastic fiber thinning was heterogeneous within the aorta of an individual patient with BAV. The association between WSS and regional histopathology was the most closely, which predominant manifested as AS and mildly aortic dilation (<4.5 cm) (Barker et al., 2018; Bollache et al., 2018).

After repeated modifications in different international guidelines on BAV aortopathy, AAR (ascending aorta replacement) might be a suitable option for patients with severe AS or AR who have suffered AVR, when diameter of aorta was >4.5 cm (Hiratzka et al., 2016; Nishimura et al., 2017). When the situation that the dilation of aortas reaches 5.1–5.5 cm and aortic dissection or aortic growth rate ≥ 0.5 cm per year is presented in family history, aortic repair operation could be recommended (Hiratzka et al., 2016). Mid-term imaging after AVR and AAR indicated that prophylactic root replacement will be unnecessary in case that aortic root is not dilated in surgery, because of unlikely aortic root enlargement later in patients (Hui et al., 2018).

## Aortic Aneurysm and Dissection: Need More Predictors and Timely Recognition

Once aortic dilation was suspected, the entire aorta and affected parts which should be considered to be at risk for aortic aneurysm or dissection. Although aorta diameter ≥40 mm was considered to be aortic dilation in the 2014 AHA guideline, risk of aortic dissection was significantly correlated with the aortic diameter. The estimated risk of aortic dissection gradually increases when diameter of the aorta becomes larger (Kim et al., 2016).

Due to aorta dynamic change with the heart contraction and diastole, aortic size alone was not enough to distinguish different pathological processes under the risk of acute complications. Other predictors about aortic wall and blood flow changes could be developed to assess the timing of surgical intervention (Bhave and Eagle, 2018). Wall shear stress, flow displacement and helicity were elevated in patients with BAV, particularly at locations of aneurysm formation (Bissell et al., 2013; Youssefi et al., 2017). The anterior region of ascending aortic aneurysm sample (the zone with lower curvature) was the most weakness and less stiff, resulting in the ascending aortic aneurysm expansion in such a region (Ferrara et al., 2018). This might provide the possible reason that patients with aortic diameter whose size do not meet surgical criteria are prone to acute aortic rupture or dissection. 4D flow MRI seemed to be a useful tool showing biphasic trend followed by dilated diameter of aneurysm. With the diameter of ascending aortic enlarged, it decreased firstly and increased. This curve has an obvious turning point as the diameter of ascending aortic was 50 mm (Guala et al., 2018). The single diameter of aortic root or ascending aortic should not be used as a basis for the possible formation of aortic dissection. The cross-sectional area to height ratio and patient – specific aneurysm wall stress analysis were recommended to predict aortic dissection (Wojnarski et al., 2015; Hiratzka et al., 2016; Xuan et al., 2018). European and American guidelines for intervention for BAV aortic aneurysms are not consistent. In ESC guideline, interventions should be recommended for patients with ascending aortic diameter ≥50 mm and risk factors (Erbel et al., 2014b; Iung, 2015). In American guidelines, for patients of BAV, the diameter of ascending aortic or sinus is larger than 5.5 cm, surgical repair should be assessed and recommended (Hiratzka et al., 2010).

As BAV with aortic aneurysm could progress to aortic dissections, timely recognition of aneurysm can be lifesaving due to the high mortality rate (Evangelista, 2016). Young adults were better treated with reimplantation of the aortic valve, as correction of annular dilation was important. The closer 2 main commissures are to 180°, the better the long-term results will be (David, 2016). For some young patients, the aortic cusps are nearly normal anatomically and morphologically, aortic valve-sparing root replacement was also an effective alternative (Ouzounian et al., 2016). In contrast, recent study provided that valve-sparing root replacement and bio-composite valve led to equivalent operative mortality and morbidity with similar midterm survival and valve durability (Esaki et al., 2017). In emergency situation, aortic valve replacement could be performed to AD patients (Erbel et al., 2014a,b). Aortic valve resuspension with supra-coronary aortic replacement could be recommended to restore aortic valve competency in most AD patients with acceptable mid-term survival (Tang et al., 2017).

## Pediatric BAV: Cannot Apply Adult Knowledge

Pediatric BAV, although similar to adult BAV, has different characteristics in disease development and treatment. According to Sievers classification, BAV morphology including type 0 (55%), type 1 (41%), and type 2 (4%) in children (Merkx et al., 2017). At an early stage, some children with well-functioning BAV have abnormalities in some parameters of diastolic function of LV and even all parameters of proximal and distal ascending aortic elasticity. However, there is no relationship between the aortic stiffness and LV diastolic impairment (Weismann et al., 2016). *Z*-scores of children seem higher at the site of annulus, sinus valsalva, sinutubular junction, and ascending aorta. Moreover, the aortas start to enlarge in BAV patients from childhood and progress as time goes on, 0.06 (Gautier) and 0.09 (Campens) units per year at the age of 5–15 years, which delivers us a signal that generalized aortopathy has come around. The elasticity of aortas increases at young age and begins to decrease with age (Verma and Siu, 2014). In some pediatric patients with BAV, aortic growth is not proportional to body growth. The sizes of sinuses of Valsalva or ascending aorta seems to be larger, which might be associated with AR or AS. BAV morphology is a important factor that could directly affect the dilation of aortic sinus. Although the dilation of aortic sinus is not existing patients who manifests as RN fusion morphology, the median *Z* score of aortic sinus is significantly higher in these who have RL fusion morphology, which is keeping in accordance with some conclusions obtained from adults (Jassal et al., 2010; Khoo et al., 2013). And the RN fusion morphology is associated with significantly greater incidence and undesirable prognosis of aortic valve stenosis and aortic insufficiency in contrast to ascending aorta (Ward et al., 2018).

For the patient with isolated, normal functioning BAV, echocardiography could be used to monitor development of BAV associated complications (Merkx et al., 2017). Due to the inaccuracy in measurement of aortic valve annular eccentricity through 2D echocardiography, therefore, 3D echocardiography could be a better choice to evaluate the AV annulus which has the shape of ellipse and keeps the same in all patients (Chamberland et al., 2015). On the contrary to adult patients, WSS seems to be not related to ascending aortic dilation in pediatric BAV patients after the valvular disease being controlled. The enlarging of aorta diameters causing the reduction of WSS, which takes place in adult BAV, will keep a long time and seems to be scarcely in pediatric BAV patients (Allen et al., 2015). Echocardiographic endpoints are measured as *z*-score, which manifests as progressive AS or AR and aortic enlargement at different levels of the aortic root. It is benign in the period of medium-term follow-up. It is suggested that the degree of AS was constant in 95% of patients and about 85% of the patients showed no increase in the degree of AR. Although the ascending aorta dilation was observed (*z*-score >2) and the incidence was less than one-fifth of the patients, the progressive dilation was not existed in annulus, Valsalva sinuses, or sinotubular junction (Spaziani et al., 2014).

Stenosis is the dominating clinical feature in younger patients and regurgitant becomes predominant when patients are stepping into adulthood. It has been suggested that regurgitant valves are always accompany with stenotic parts that is the result of commissural fusion, which restricts the motion of the leaflet in pediatric patients (Siddiqui et al., 2013). There are six types of the dilated aorta in children with BAV including the normal shape (S1), the enlarged ascending aorta (S2), the effacement of the sinotubular ridge (S3), the Marfan-like (S4), the enlarged sinus of Valsalva and ascending aorta (S5), the normal annulus, and proximal sinus of Valsalva, enlarged distal sinus of Valsalva, sinotubular ridge, and ascending aorta (S6). S2 and S3 are more presented than other types. S3 becomes the dominating type with aortic dilation becoming more significant. Although some patients with S2 or S3 BAV shape have no aortic dilation, the ascending aorta was larger compared with normal aorta, which also suggested that the process of abnormal dilation might have already begun (Mart and McNerny, 2013).

The effect of surgical repair in pediatric BAV patients is excellent, especially no patches are attached. The primary repair is recommended because enduring results can be obtained with simple procedures. It is suggested that some patients underwent the surgery of bicuspid valve repairment without addition any patch material and there is no adverse events for up to 10 years because the valves used in the surgery is similar to the native valves. Up to now, compared with an extensive debridement of the valve, it is hard to identify the patients who will have a better prognosis. The ross procedure is recommended to carry out in adult age because a quarter of the patients need to replace their autografts within 2 decades, which we hope to create a situation that the root of autograft is reinforced more easily (Siddiqui et al., 2013). Aortic incompetence (AI) is the main reason for aortic valve balloon valvuloplasty surgery after BAV (Balmer et al., 2004). The degree of AI is closely related to the ratio of balloon/annulus, but not related to the valve morphology. Balloon valvuloplasty is associated with the decreasing mortality and could reduce acute postoperative complications, which root in the decreasing ratio of balloon/annulus that is a well-known risk factor to increase the acute complications (Knirsch et al., 2008).

## Marfan and Loeys-Dietz Syndrome: Similar With the Ascending Aortic Dilatation in BAV

Bicuspid aortic valve with aortic dilation should be distinguished from other symptom-similar diseases, such as Loeys–Dietz syndrome and Marfan syndrome. The syndrome of Marfan is characterized by heritable connective tissue lesion caused by the mutation of FBN1 gene among 90% patients and involved in multiple organs including cardiac and valve (Baumgartner et al., 2010). Marfan syndrome has the higher risk of dissection and recurrent aneurysm than BAV with aortic dilation. For patients who have haploinsufficient FBN1 mutations, angiotensin II receptor inhibitor might reduce aortic root dilatation rate (Franken et al., 2015). β-Blockers is used to reduce the aortic dilation and improve the rate of survival (Engelfriet and Mulder, 2007). It has been suggested that patients need to have surgery when the maximal diameter of aortic root is more than 50 mm or other parts of the aorta >50 mm or dilation is progressive according to ESC guidelines that about the management of congenital heart disease in adult patients. In some cases, although the aortic root diameter of patients is between 46 and 50 mm, not reaching the criterion 50 mm, they still need surgery because of progressive dilation > 2 mm/year (Baumgartner et al., 2010; Erbel et al., 2014a,b). And the JCS guideline recommend a more rigid criterion for surgery, maximal aortic diameter ≥45 mm or >40 mm but with positive family history (Joint Working Group, 2013). When Marfan syndrome and pregnancy are co-occurrences in one women, it is suggested to make the root part of the aorta and ascending aorta replaced if the diameter > 40 mm according to AHA; however, the aortic root aneurysm ≥45 mm is the criterion for surgery according to ESC (Hiratzka et al., 2010). It is critical to perform the surgery with the experienced hands, which could reduce the risk of combining aortic valve and ascending aorta replacement (Gott et al., 1999). Valve-sparing operations or remodeling of the aortic root should be taken into consideration if the valves with normal anatomically (Kallenbach et al., 2007).

Few BAV patients were identified in Marfan syndrome patients. According to a study regarding association of Marfan syndrome and BAV, it is demonstrated that the association between the two disorders seems to cause a higher percentage of thoracic aortic aneurysm requiring surgical intervention (Nistri et al., 2012).

Loeys–Dietz syndrome, an autosomal-dominant connective tissue disorder, has different types based on gene mutations in the TGFBR1 (type 1), TGFBR2 (type 2), SMAD3 (type 3), and TGFB2 (type 4) (MacCarrick et al., 2014). Loeys–Dietz syndrome has the characteristics of rapidly progressive aortic aneurysm and aortic dissection and cerebral hemorrhages are major causes of death (Loeys et al., 2006). Hence, echocardiography monitoring is recommended as a useful diagnosis method to this syndrome (Van Hemelrijk et al., 2010). If some variations are observed including aortic root dimension >40 mm or the expansion of aortic root >0.5 cm over 1 year or the dimensions of the ascending aorta >40 mm in adults and aortic annulus is 20–22 mm or the expansion of aorta > 0.5 cm over 1 year or severe craniofacial features or positive family history about aggressive aortic disease in children, it is time for surgical intervention (Renard et al., 2013). In American guideline, the aorta need to be repaired in these situations including BAV with Loeys–Dietz syndrome, the diameter of aorta > 4.2 cm by transoesophageal echocardiogram (internal diameter) with mutations in TGFBR1 or TGFBR2 or the external diameter > 4.4 cm by CT or MRI (Hiratzka et al., 2010).

The surgery of valve-sparing seems to be prohibited in the situation of extensive calcification or dysfunction in BAV patients (Kasar et al., 2018). But the replacement surgery of valve-sparing aortic root could be recommended in experienced hands for children (Cleuziou et al., 2010). It is necessary to perform the aortic root repair surgery when the maximal dimension of aortic root reaches 40 mm in adults with LDS type 1 or 2 (MacCarrick et al., 2014). So far, there is no clear and comprehensive guideline to introduce the therapy on Loeys–Dietz syndrome.

## New Technique: Developing Minimally Invasive Technique

Many of the aortic valve replacements used clinically are not alive and ROSS procedure is an exception. In order to preserve the anatomical and functional integrity of the aortic root, ROSS procedure utilizes pulmonary autograft instead of lesions aortic valve (Mazine et al., 2018). ROSS procedure has good hemodynamic performance during activity and the lower gradient of normal aortic valve function at rest (Puranik et al., 2010). Patients who undergo ROSS surgery have a higher quality of life than patients who undergo mechanical valves (Zacek et al., 2016). ROSS has been improved in past several decades. At first, pulmonary autograft transplantation with the sub coronary and root replacement techniques translated into a full root replacement technique (Sievers et al., 2016). To prevent from postoperative expansion, prosthetic Dacron graft could be used to strengthening the pulmonary autograft (Carrel and Kadner, 2016). However, ROSS procedure has not been adopted worldwide with underlying reasons as operative risk and technical complexity. As a result, it increases possible failure of valves stability for long and complexity of the second intervention surgery (Reece et al., 2014). ROSS procedure is more suitable for younger patients or middle-aged (<50 years old) patients or prosthetic AVR in young patients with aortic insufficiency (Ouzounian et al., 2017). 85% of BAV patients, whose median age is 40 years old, could be freedom from reoperation at 20 years with a follow-up of 9 years (Poh et al., 2018).

In spite of valve replacement is standard therapy now, BAV patients with normal cusps might be recommended to have valve repair (Zeeshan et al., 2018). For young patients, the aortic annulus does not match the adult-sized prosthesis and need normal root structure to grow. Hence, it is necessary to retain native aortic root in valve repair operation. Although cusp extension has no newer techniques, it seems unlikely to be avoided for young people as a part of aortic valve repair. Compared with BAV repair in children with median age 12.6 years by tricuspidization with cusp extension (TCE), the stability of valve function after ROSS procedure seems to be higher (McMullan et al., 2007). The other repair techniques used in the past have commissural suture, cusp procedures and aortic root procedures. The cusp procedures contain cusp resection, patching, commissurotomy, debridement and free-margin plication. The aortic root procedures include root remodeling, resuspension and reimplantation. Recently, figure-of-8 suspensory sutures (Svensson) or Cabrol commissuroplasty are carried out in aortic valve repair. The point of suspensory sutures in Svensson operation is the lip of cusps at 3–4 mm above the commissure (Idrees et al., 2015). The latter is plication at center and 2 commissural stitches. Compare the outcomes of these techniques, Svensson technique has lower rate of reoperation and better durability for long, as well as valve resuspension, root repair and replacement (Svensson et al., 2014). However, some of cusp repair procedures such as commissuroplasty, commissural sutures and plication might be related to higher rate of reoperation (Jasinski et al., 2015). Kari technique could correct prolapse combined with valve-sparing aortic root replacement and cusp repair. 90% of the patients avoided reoperation at 6 years and 100% avoided higher than grade 2 aortic regurgitation (Kari et al., 2013).

Ozaki technique was first reported in 2014 and use autologous pericardium to replace tricuspid. The pericardial cusp was excised at least 7 cm × 8 cm, and was sutured to the annulus (Ozaki et al., 2014). In 850 patients with average age of 71 years who have undergone Ozaki technique, 85.9% have the second operation, 4.2% have moderate AR and nobody had additional valve replacement. There was no conversion to a prosthetic valve replacement. The midterm outcome seemed to be satisfactory (Reuthebuch et al., 2018). For younger patients, Ozaki technique might have an advantage because it required only Aspirin, rather than antivitamin K. The other one was that it did not need enlarge aortic root for patients with small annulus. Given the benefits of Ozaki technique, minimally Invasive Ozaki’s Procedure was carried out in nine patients. First, the minimally invasive procedure could provide clear surgical field by smaller incision to collect pericardium and reduce damage to the heart or risk of hemostasis. In addition, patients could be freedom from ventilation earlier, reduce ICU and hospital stay after operation. Thus, upper ministernotomy combined with Ozaki procedure might be a feasible approach instead of full sternotomy (Nguyen et al., 2018). However, Ozaki technique or combined ministernotomy require longer follow-up outcomes in young adults.

## Future Directions

**FIGURE 2:**
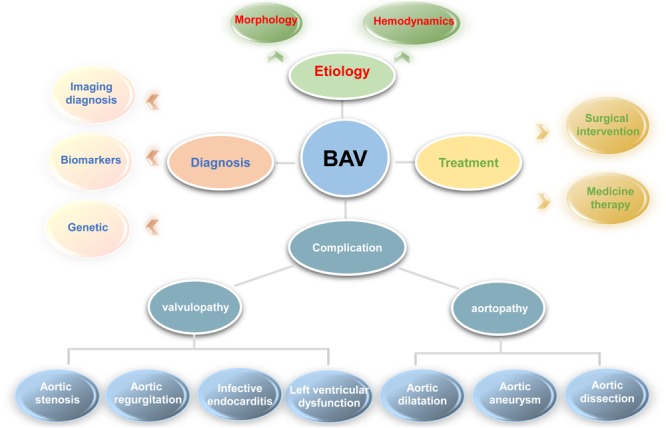
For better understanding the problems involved in bicuspid aortic valve, a clear diagram was provided. In general, it includes the etiology (morphology and abnormal hemodynamics described in complication section), diagnosis (imaging, biomarker and genetic diagnosis), treatment (medicine and surgical treatment), and various complications involved in bicuspid aortic valve.

Bicuspid aortic valve is a general geneogenous heart defects and burden from BAV is more significant than other congenital cardiac lesions around the word. Despite many emerging studies focus on the pathogenesis, diagnosis and treatment of BAV as shown in Figure 2, our understanding of the disease is still incomplete and many questions remain need to be illuminated in future studies.

Gene mutations polymorphism and aberrant changes in hemodynamics have been thought to be the two main aspects of BAV pathogenesis. Although it has been reported that many genes and cellular signaling pathway were involved in malformations of valves, none has been confirmed to play central role. This restricts the clinical translational applications of research findings to patients timely. Hemodynamics which is affected by malformations of the valves, might not be reversed by preventive surgical intervention. To date, it is necessary to perfume early biomarkers test and multiple imaging diagnosis including ultrasound, cardiac CT and 4D MRI. Although given the weight of evidence about aberrant biomarkers in BAV patients, it still needs to be clarified the importance of these biomarkers when they are appeared in patients’ samples. Besides, the relationship between these biomarkers and severity or prognosis of BAV need to be explored further. Therefore, specificity and sensibility of biomarkers are relative insufficiency, and could not provide accurate information for doctors. Combined imaging diagnosis seems to be the only effective method to evaluate valve morphology, function, hemodynamics and complications, which present evidence for timely surgical intervention.

Although medicine treatment has not achieved obvious effect in BAV patients, gene targeted delivery or therapy might be a promising treatment to prevent BAV and its complications according to widely genetic research. So far, surgical intervention is the only efficient way in the management of patients. The threshold of surgery, aortic diameter, has been modified repeatedly in the international guidelines, but it is inadequate to draw a perfect heal scheme. Although aortic valve replacement is the major surgical therapy recently, other surgical techniques still need to be explored and tried. It would be better if they could be combined with invasive techniques in the future. Since the development of BAV is affected by multiple factors as mentioned before, optimal surgical intervention and comprehensive treatment options should be individualized to the patients in the future.

## Author Contributions

TL performed literature review and drafted the article. MX, QL, YL, LF, and LZ contributed to the revision and edition of the manuscript. WD collected the articles. JW revised and critically appraised the manuscript for intellectual content. All authors read and approved the final manuscript.

## Conflict of Interest Statement

The authors declare that the research was conducted in the absence of any commercial or financial relationships that could be construed as a potential conflict of interest.

## References

[B1] Allen B. D. van Ooij P. Barker A. J. Carr M. Gabbour M. Schnell S. (2015). Thoracic aorta 3D hemodynamics in pediatric and young adult patients with bicuspid aortic valve. *J. Magn. Reson. Imaging* 42 954–963. 10.1002/jmri.24847 25644073 PMC4511732

[B2] Ambale-Venkatesh B. Lima J. A. (2015). Cardiac MRI: a central prognostic tool in myocardial fibrosis. *Nat. Rev. Cardiol.* 12 18–29. 10.1038/nrcardio.2014.159 25348690

[B3] Arangalage D. Laredo M. Ou P. Brochet E. Cimadevilla C. Enriquez-Sarano M. (2018). Anatomic characterization of the aortic root in patients with bicuspid and tricuspid aortic valve stenosis: does fusion of doppler-echocardiography and computed tomography resolve discordant severity grading? *JACC Cardiovasc. Imaging* 10.1016/j.jcmg.2018.04.013 [Epub ahead of print]. 29909115

[B4] Balistreri C. R. Crapanzano F. Schirone L. Allegra A. Pisano C. Ruvolo G. (2018). Deregulation of Notch1 pathway and circulating endothelial progenitor cell (EPC) number in patients with bicuspid aortic valve with and without ascending aorta aneurysm. *Sci. Rep.* 8:13834. 10.1038/s41598-018-32170-2 30218064 PMC6138685

[B5] Balmer C. Beghetti M. Fasnacht M. Friedli B. Arbenz U. (2004). Balloon aortic valvoplasty in paediatric patients: progressive aortic regurgitation is common. *Heart* 90 77–81. 10.1136/heart.90.1.77 14676250 PMC1768038

[B6] Barker A. J. Markl M. Fedak P. (2018). Assessing wall stresses in bicuspid aortic valve-associated aortopathy: forecasting the perfect storm? *J. Thorac. Cardiovasc. Surg.* 156 471–472. 10.1016/j.jtcvs.2018.03.092 29666014

[B7] Baumgartner H. Bonhoeffer P. De Groot N. M. de Haan F. Deanfield J. E. Galie N. (2010). ESC Guidelines for the management of grown-up congenital heart disease (new version 2010). *Eur. Heart J.* 31 2915–2957. 10.1093/eurheartj/ehq249 20801927

[B8] Bavaria J. E. Komlo C. M. Rhode T. Vallabhajosyula P. (2013). Can the bicuspid aortic valve be spared? The con position, with caveats and nuances. *Tex. Heart Inst. J.* 40 544–546. 24391315 PMC3853810

[B9] Ben-Dor I. Stewart A. (2017). A cautionary tale of 2 leaflets: TAVR in bicuspid aortic valve stenosis. *J. Am. Coll. Cardiol.* 69 2590–2591. 10.1016/j.jacc.2017.03.573 28545632

[B10] Bhave N. M. Eagle K. A. (2018). “Much More Than a Tube”: the aneurysmal ascending aorta as a dynamic entity. *JACC Cardiovasc. Imaging* 10.1016/j.jcmg.2018.04.006 [Epub ahead of print]. 29778847

[B11] Bissell M. M. Hess A. T. Biasiolli L. Glaze S. J. Loudon M. Pitcher A. (2013). Aortic dilation in bicuspid aortic valve disease: flow pattern is a major contributor and differs with valve fusion type. *Circ. Cardiovasc. Imaging* 6 499–507. 10.1161/CIRCIMAGING.113.000528 23771987 PMC3859916

[B12] Bjorck H. M. Du L. Pulignani S. Paloschi V. Lundstromer K. Kostina A. S. (2018). Altered DNA methylation indicates an oscillatory flow mediated epithelial-to-mesenchymal transition signature in ascending aorta of patients with bicuspid aortic valve. *Sci. Rep.* 8:2777. 10.1038/s41598-018-20642-4 29426841 PMC5807320

[B13] Boe B. A. Zampi J. D. Kennedy K. F. Jayaram N. Porras D. Foerster S. R. (2017). Acute success of balloon aortic valvuloplasty in the current Era: a national cardiovascular data registry study. *JACC Cardiovasc. Interv.* 10 1717–1726. 10.1016/j.jcin.2017.08.001 28882282

[B14] Bollache E. Guzzardi D. G. Sattari S. Olsen K. E. Di Martino E. S. Malaisrie S. C. (2018). Aortic valve-mediated wall shear stress is heterogeneous and predicts regional aortic elastic fiber thinning in bicuspid aortic valve-associated aortopathy. *J. Thorac. Cardiovasc. Surg.* 156 2112–2120.e2. 10.1016/j.jtcvs.2018.05.095 30060930 PMC6242768

[B15] Boonyasirinant T. Rajiah P. Flamm S. D. (2018). Abnormal aortic stiffness in patients with bicuspid aortic valve: phenotypic variation determined by magnetic resonance imaging. *Int. J. Cardiovasc. Imaging* 10.1007/s10554-018-1433-y [Epub ahead of print]. 30187149

[B16] Braverman A. C. Guven H. Beardslee M. A. Makan M. Kates A. M. Moon M. R. (2005). The bicuspid aortic valve. *Curr. Probl. Cardiol.* 30 470–522. 10.1016/j.cpcardiol.2005.06.002 16129122

[B17] Broch K. de Marchi S. F. Massey R. Hisdal J. Aakhus S. Gullestad L. (2017). Left ventricular contraction pattern in chronic aortic regurgitation and preserved ejection fraction: simultaneous stress-strain analysis by three-dimensional echocardiography. *J. Am. Soc. Echocardiogr.* 30 422–430. 10.1016/j.echo.2016.11.012 28065583

[B18] Carrel T. Kadner A. (2016). Long-term clinical and imaging follow-up after reinforced pulmonary autograft ross procedure. *Semin. Thorac. Cardiovasc. Surg. Pediatr. Card. Surg. Annu.* 19 59–62. 10.1053/j.pcsu.2015.11.005 27060045

[B19] Chamberland C. R. Sugeng L. Abraham S. Li F. Weismann C. G. (2015). Three-dimensional evaluation of aortic valve annular shape in children with bicuspid aortic valves and/or aortic coarctation compared with controls. *Am. J. Cardiol.* 116 1411–1417. 10.1016/j.amjcard.2015.07.063 26375172

[B20] Chambers J. B. (2018). Antibiotic prophylaxis against infective endocarditis: widening the net? *J. Am. Coll. Cardiol.* 71 2741–2743. 10.1016/j.jacc.2018.04.021 29903347

[B21] Chen J. Lu S. Hu K. Yang Z. Pan S. Hong T. (2017). Clinical characteristics and surgical treatment of infective endocarditis with bicuspid aortic valve. *Int. Heart J.* 58 220–224. 10.1536/ihj.16-284 28367850

[B22] Clavel M. A. Malouf J. Messika-Zeitoun D. Araoz P. A. Michelena H. I. Enriquez-Sarano M. (2015). Aortic valve area calculation in aortic stenosis by CT and Doppler echocardiography. *JACC Cardiovasc. Imaging* 8 248–257. 10.1016/j.jcmg.2015.01.009 25772832

[B23] Cleuziou J. Eichinger W. B. Schreiber C. Lange R. (2010). Aortic root replacement with re-implantation technique in an infant with Loeys-Dietz syndrome and a bicuspid aortic valve. *Pediatr. Cardiol.* 31 117–119. 10.1007/s00246-009-9541-z 19784694

[B24] Cotrufo M. Della C. A. De Santo L. S. Quarto C. De Feo M. Romano G. (2005). Different patterns of extracellular matrix protein expression in the convexity and the concavity of the dilated aorta with bicuspid aortic valve: preliminary results. *J. Thorac. Cardiovasc. Surg.* 130 504–511. 10.1016/j.jtcvs.2005.01.016 16077420

[B25] Dargis N. Lamontagne M. Gaudreault N. Sbarra L. Henry C. Pibarot P. (2016). Identification of gender-specific genetic variants in patients with bicuspid aortic valve. *Am. J. Cardiol.* 117 420–426. 10.1016/j.amjcard.2015.10.058 26708639

[B26] David T. E. (2016). Aortic valve sparing in different aortic valve and aortic root conditions. *J. Am. Coll. Cardiol.* 68 654–664. 10.1016/j.jacc.2016.04.062 27491910

[B27] de Kerchove L. Vismara R. Mangini A. Fiore G. B. Price J. Noirhomme P. (2012). In vitro comparison of three techniques for ventriculo-aortic junction annuloplasty. *Eur. J. Cardiothorac. Surg.* 41 1117–1123; discussion 1123–1124. 10.1093/ejcts/ezr237 22228850

[B28] Detaint D. Michelena H. I. Nkomo V. T. Vahanian A. Jondeau G. Sarano M. E. (2014). Aortic dilatation patterns and rates in adults with bicuspid aortic valves: a comparative study with Marfan syndrome and degenerative aortopathy. *Heart* 100 126–134. 10.1136/heartjnl-2013-304920 24254191

[B29] Disha K. Dubslaff G. Rouman M. Fey B. Borger M. A. Barker A. J. (2017). Evidence of subannular and left ventricular morphological differences in patients with bicuspid versus tricuspid aortic valve stenosis: magnetic resonance imaging-based analysis. *Interact. Cardiovasc. Thorac. Surg.* 24 369–376. 10.1093/icvts/ivw363 28040769 PMC6283034

[B30] Donati F. Myerson S. Bissell M. M. Smith N. P. Neubauer S. Monaghan M. J. (2017). Beyond Bernoulli: improving the accuracy and precision of noninvasive estimation of peak pressure drops. *Circ. Cardiovasc. Imaging* 10:e005207. 10.1161/CIRCIMAGING.116.005207 28093412 PMC5265685

[B31] Du J. Zheng R. Xiao F. Zhang S. He K. Zhang J. (2017). Downregulated MicroRNA-195 in the bicuspid aortic valve promotes calcification of valve interstitial cells via targeting SMAD7. *Cell. Physiol. Biochem.* 44 884–896. 10.1159/000485356 29176317

[B32] Dweck M. R. Joshi S. Murigu T. Gulati A. Alpendurada F. Jabbour A. (2012). Left ventricular remodeling and hypertrophy in patients with aortic stenosis: insights from cardiovascular magnetic resonance. *J. Cardiovasc. Magn. Reson.* 14:50. 10.1186/1532-429X-14-50 22839417 PMC3457907

[B33] Dyverfeldt P. Bissell M. Barker A. J. Bolger A. F. Carlhall C. J. Ebbers T. (2015). 4D flow cardiovascular magnetic resonance consensus statement. *J. Cardiovasc. Magn. Reson.* 17:72. 10.1186/s12968-015-0174-5 26257141 PMC4530492

[B34] El K. G. Glineur D. Rubay J. Verhelst R. D’Acoz Y. Poncelet A. (2005). Functional classification of aortic root/valve abnormalities and their correlation with etiologies and surgical procedures. *Curr. Opin. Cardiol.* 20 115–121. 10.1097/01.hco.0000153951.31887.a6 15711197

[B35] Engelfriet P. Mulder B. (2007). Is there benefit of beta-blocking agents in the treatment of patients with the Marfan syndrome? *Int. J. Cardiol.* 114 300–302. 10.1016/j.ijcard.2006.01.025 16766056

[B36] Erbel R. Aboyans V. Boileau C. Bossone E. Bartolomeo R. D. Eggebrecht H. (2014a). 2014 ESC Guidelines on the diagnosis and treatment of aortic diseases: document covering acute and chronic aortic diseases of the thoracic and abdominal aorta of the adult. The task force for the diagnosis and treatment of aortic diseases of the European society of cardiology (ESC). *Eur. Heart J.* 35 2873–2926. 10.1093/eurheartj/ehu281 25173340

[B37] Erbel R. Aboyans V. Boileau C. Bossone E. Di Bartolomeo R. Eggebrecht H. (2014b). [2014 ESC Guidelines on the diagnosis and treatment of aortic diseases]. *Kardiol. Pol.* 72 1169–1252. 10.5603/KP.2014.0225 25524604

[B38] Esaki J. Leshnower B. G. Binongo J. N. Lasanajak Y. McPherson L. Halkos M. E. (2017). Clinical outcomes of the David V valve-sparing root replacement compared with bioprosthetic valve-conduits for aortic root aneurysms. *Ann. Thorac. Surg.* 103 1824–1832. 10.1016/j.athoracsur.2016.09.055 27964919

[B39] Evangelista A. (2016). Familial nonsyndromic thoracic aortic aneurysms: unraveling the mystery and defining long-term outcome. *J. Am. Coll. Cardiol.* 67 627–629. 10.1016/j.jacc.2015.11.038 26868686

[B40] Farag E. S. van Ooij P. Planken R. N. Dukker K. de Heer F. Bouma B. J. (2018). Aortic valve stenosis and aortic diameters determine the extent of increased wall shear stress in bicuspid aortic valve disease. *J. Magn. Reson. Imaging* 48 522–530. 10.1002/jmri.25956 29451963 PMC6099246

[B41] Ferrara A. Totaro P. Morganti S. Auricchio F. (2018). Effects of clinico-pathological risk factors on in-vitro mechanical properties of human dilated ascending aorta. *J. Mech. Behav. Biomed. Mater.* 77 1–11. 10.1016/j.jmbbm.2017.08.032 28886508

[B42] Forte A. Bancone C. Cobellis G. Buonocore M. Santarpino G. Fischlein T. (2017). A possible early biomarker for bicuspid aortopathy: circulating transforming growth factor beta-1 to soluble Endoglin ratio. *Circ. Res.* 120 1800–1811. 10.1161/CIRCRESAHA.117.310833 28420669

[B43] Franken R. den Hartog A. W. Radonic T. Micha D. Maugeri A. van Dijk F. S. (2015). Beneficial outcome of losartan therapy depends on type of FBN1 mutation in Marfan syndrome. *Circ. Cardiovasc. Genet.* 8 383–388. 10.1161/CIRCGENETICS.114.000950 25613431

[B44] Freeze S. L. Landis B. J. Ware S. M. Helm B. M. (2016). Bicuspid aortic valve: a review with recommendations for genetic counseling. *J. Genet. Couns.* 25 1171–1178. 10.1007/s10897-016-0002-6 27550231 PMC5141520

[B45] Gamaza-Chulian S. Diaz-Retamino E. Camacho-Freire S. Ruiz-Fernandez D. Gutierrez-Barrios A. Oneto-Otero J. (2017). Acceleration time and ratio of acceleration time to ejection time in aortic stenosis: new echocardiographic diagnostic parameters. *J. Am. Soc. Echocardiogr.* 30 947–955. 10.1016/j.echo.2017.06.001 28781116

[B46] Gaztanaga J. Paruchuri V. Elias E. Wilner J. Islam S. Sawit S. (2016). Prognostic value of late gadolinium enhancement in nonischemic cardiomyopathy. *Am. J. Cardiol.* 118 1063–1068. 10.1016/j.amjcard.2016.06.059 27614850

[B47] Gharibeh L. Komati H. Bosse Y. Boodhwani M. Heydarpour M. Fortier M. (2018). GATA6 regulates aortic valve remodeling and its haploinsufficiency leads to RL-type bicuspid aortic valve. *Circulation* 138 1025–1038. 10.1161/CIRCULATIONAHA.117.029506 29567669 PMC6151169

[B48] Girdauskas E. Disha K. Espinoza A. Misfeld M. Reichenspurner H. Borger M. A. (2017). Mitral regurgitation after previous aortic valve surgery for bicuspid aortic valve insufficiency. *J. Cardiovasc. Surg.* 58 473–480. 10.23736/S0021-9509 27012929

[B49] Giusti B. Sticchi E. De Cario R. Magi A. Nistri S. Pepe G. (2017). Genetic bases of bicuspid aortic valve: the contribution of traditional and high-throughput sequencing approaches on research and diagnosis. *Front. Physiol.* 8:612. 10.3389/fphys.2017.00612 28883797 PMC5573733

[B50] Gott V. L. Greene P. S. Alejo D. E. Cameron D. E. Naftel D. C. Miller D. C. (1999). Replacement of the aortic root in patients with Marfan’s syndrome. *N. Engl. J. Med.* 340 1307–1313. 10.1056/NEJM199904293401702 10219065

[B51] Goudot G. Mirault T. Rossi A. Zarka S. Albuisson J. Achouh P. (2018). Segmental aortic stiffness in patients with bicuspid aortic valve compared with first-degree relatives. *Heart* 105 130–136. 10.1136/heartjnl-2018-313232 30077994

[B52] Gu J. Chen Y. Zhang H. Meng W. Hu J. Zhang E. (2014). Multimodality images of a giant blood cyst originating from the bicuspid aortic valve. *Circulation* 130 e165–e166. 10.1161/CIRCULATIONAHA.114.012465 25366838

[B53] Guala A. Rodriguez-Palomares J. Dux-Santoy L. Teixido-Tura G. Maldonado G. Galian L. (2018). Influence of aortic dilation on the regional aortic stiffness of bicuspid aortic valve assessed by 4-dimensional flow cardiac magnetic resonance: comparison with Marfan syndrome and degenerative aortic aneurysm. *JACC Cardiovasc. Imaging* 10.1016/j.jcmg.2018.03.017 [Epub ahead of print]. 29778849

[B54] Helgadottir A. Thorleifsson G. Gretarsdottir S. Stefansson O. A. Tragante V. Thorolfsdottir R. B. (2018). Genome-wide analysis yields new loci associating with aortic valve stenosis. *Nat. Commun.* 9:987. 10.1038/s41467-018-03252-6 29511194 PMC5840367

[B55] Hirata Y. Aoki H. Shojima T. Takagi K. Takaseya T. Akasu K. (2018). Activation of the AKT pathway in the ascending aorta with bicuspid aortic valve. *Circ. J.* 82 2485–2492. 10.1253/circj.CJ-17-1465 30089758

[B56] Hiratzka L. F. Bakris G. L. Beckman J. A. Bersin R. M. Carr V. F. Casey D. J. (2010). 2010 ACCF/AHA/AATS/ACR/ASA/SCA/SCAI/SIR/STS/SVM guidelines for the diagnosis and management of patients with thoracic aortic disease: executive summary. A report of the American College of Cardiology Foundation/American Heart Association Task Force on Practice Guidelines, American Association for Thoracic Surgery, American College of Radiology, American Stroke Association, Society of Cardiovascular Anesthesiologists, Society for Cardiovascular Angiography and Interventions, Society of Interventional Radiology, Society of Thoracic Surgeons, and Society for Vascular Medicine. *Catheter. Cardiovasc. Interv.* 76 E43–E86. 10.1002/ccd.22537 20687249

[B57] Hiratzka L. F. Creager M. A. Isselbacher E. M. Svensson L. G. Nishimura R. A. Bonow R. O. (2016). Surgery for aortic dilatation in patients with bicuspid aortic valves: a statement of clarification from the American college of cardiology/American heart association task force on clinical practice guidelines. *Circulation* 133 680–686. 10.1161/CIR.0000000000000331 26637530

[B58] Hui S. K. Fan C. S. Christie S. Feindel C. M. David T. E. Ouzounian M. (2018). The aortic root does not dilate over time after replacement of the aortic valve and ascending aorta in patients with bicuspid or tricuspid aortic valves. *J. Thorac. Cardiovasc. Surg.* 156 5–13. 10.1016/j.jtcvs.2018.02.094 29656818

[B59] Idrees J. J. Roselli E. E. Arafat A. Johnston D. R. Svensson L. G. Sabik J. R. (2015). Outcomes after repair or replacement of dysfunctional quadricuspid aortic valve. *J. Thorac. Cardiovasc. Surg.* 150 79–82. 10.1016/j.jtcvs.2015.03.019 25896461

[B60] Iung B. (2015). New ESC guidelines: aortic disease. *Heart* 101 421–423. 10.1136/heartjnl-2014-306777 25550317

[B61] Jasinski M. J. Gocol R. Malinowski M. Hudziak D. Duraj P. Deja M. A. (2015). Predictors of early and medium-term outcome of 200 consecutive aortic valve and root repairs. *J. Thorac. Cardiovasc. Surg.* 149 123–129. 10.1016/j.jtcvs.2014.08.057 25439785

[B62] Jassal D. S. Bhagirath K. M. Tam J. W. Sochowski R. A. Dumesnil J. G. Giannoccaro P. J. (2010). Association of Bicuspid aortic valve morphology and aortic root dimensions: a substudy of the aortic stenosis progression observation measuring effects of rosuvastatin (ASTRONOMER) study. *Echocardiography* 27 174–179. 10.1111/j.1540-8175.2009.00993.x 19725842

[B63] Jilaihawi H. Chen M. Webb J. Himbert D. Ruiz C. E. Rodes-Cabau J. (2016). A bicuspid aortic valve imaging classification for the TAVR Era. *JACC Cardiovasc. Imaging* 9 1145–1158. 10.1016/j.jcmg.2015.12.022 27372022

[B64] Jilaihawi H. Kashif M. Fontana G. Furugen A. Shiota T. Friede G. (2012). Cross-sectional computed tomographic assessment improves accuracy of aortic annular sizing for transcatheter aortic valve replacement and reduces the incidence of paravalvular aortic regurgitation. *J. Am. Coll. Cardiol.* 59 1275–1286. 10.1016/j.jacc.2011.11.045 22365424

[B65] Joint Working Group (2013). Guidelines for diagnosis and treatment of aortic aneurysm and aortic dissection (JCS 2011): digest version. *Circ. J.* 77 789–828. 10.1253/circj.CJ-66-0057 23412710

[B66] Kallenbach K. Baraki H. Khaladj N. Kamiya H. Hagl C. Haverich A. (2007). Aortic valve-sparing operation in Marfan syndrome: what do we know after a decade? *Ann. Thorac. Surg.* 83 S764–S768. 10.1016/j.athoracsur.2006.10.097 17257923

[B67] Kari F. A. Liang D. H. Kvitting J. P. Stephens E. H. Mitchell R. S. Fischbein M. P. (2013). Tirone David valve-sparing aortic root replacement and cusp repair for bicuspid aortic valve disease. *J. Thorac. Cardiovasc. Surg.* 145 S35–S40. 10.1016/j.jtcvs.2012.11.043 23260433

[B68] Kasar T. Gezdirici A. Ayyildiz P. Haydin S. Guzeltas A. (2018). Valve-sparing aortic root replacement in Loeys-Dietz syndrome and a novel mutation in TGFBR2. *Anatol. J. Cardiol.* 19 74–77. 10.14744/AnatolJCardiol.2017.7911 29339704 PMC5864794

[B69] Kerstjens-Frederikse W. S. van de Laar I. M. Vos Y. J. Verhagen J. M. Berger R. M. Lichtenbelt K. D. (2016). Cardiovascular malformations caused by NOTCH1 mutations do not keep left: data on 428 probands with left-sided CHD and their families. *Genet. Med.* 18 914–923. 10.1038/gim.2015.193 26820064

[B70] Khoo C. Cheung C. Jue J. (2013). Patterns of aortic dilatation in bicuspid aortic valve-associated aortopathy. *J. Am. Soc. Echocardiogr.* 26 600–605. 10.1016/j.echo.2013.02.017 23562085

[B71] Kim J. B. Spotnitz M. Lindsay M. E. MacGillivray T. E. Isselbacher E. M. Sundt T. R. (2016). Risk of aortic dissection in the moderately dilated ascending aorta. *J. Am. Coll. Cardiol.* 68 1209–1219. 10.1016/j.jacc.2016.06.025 27609684

[B72] Knirsch W. Berger F. Harpes P. Kretschmar O. (2008). Balloon valvuloplasty of aortic valve stenosis in childhood: early and medium term results. *Clin. Res. Cardiol.* 97 587–593. 10.1007/s00392-008-0655-8 18347766

[B73] Komiya T. (2015). Aortic valve repair update. *Gen. Thorac. Cardiovasc. Surg.* 63 309–319. 10.1007/s11748-015-0523-1 25652725

[B74] Kong W. Regeer M. V. Poh K. K. Yip J. W. van Rosendael P. J. Yeo T. C. (2018). Inter-ethnic differences in valve morphology, valvular dysfunction, and aortopathy between Asian and European patients with bicuspid aortic valve. *Eur. Heart J.* 39 1308–1313. 10.1093/eurheartj/ehx562 29029058

[B75] Kong W. K. Delgado V. Poh K. K. Regeer M. V. Ng A. C. McCormack L. (2017). Prognostic implications of raphe in bicuspid aortic valve anatomy. *JAMA Cardiol.* 2 285–292. 10.1001/jamacardio.2016.5228 28052146

[B76] Krieger E. V. Hung J. (2018). Bicuspid aortic valve type: it takes two. *Heart* 104 544–545. 10.1136/heartjnl-2017-312133 28954828

[B77] Laforest B. Andelfinger G. Nemer M. (2011). Loss of Gata5 in mice leads to bicuspid aortic valve. *J. Clin. Invest.* 121 2876–2887. 10.1172/JCI44555 21633169 PMC3223824

[B78] Lee S. Y. Shim C. Y. Hong G. R. Seo J. Cho I. Cho I. J. (2015). Association of aortic phenotypes and mechanical function with left ventricular diastolic function in subjects with normally functioning bicuspid aortic valves and comparison to subjects with tricuspid aortic valves. *Am. J. Cardiol.* 116 1547–1554. 10.1016/j.amjcard.2015.08.017 26409638

[B79] Lehoux S. Tedgui A. (2003). Cellular mechanics and gene expression in blood vessels. *J. Biomech.* 36 631–643. 10.1016/S0021-9290(02)00441-4 12694993

[B80] Li Y. Wei X. Zhao Z. Liao Y. He J. Xiong T. (2017). Prevalence and complications of bicuspid aortic valve in Chinese according to echocardiographic database. *Am. J. Cardiol.* 120 287–291. 10.1016/j.amjcard.2017.04.025 28532768

[B81] Liu X. He Y. Zhu Q. Gao F. He W. Yu L. (2018). Supra-annular structure assessment for self-expanding transcatheter heart valve size selection in patients with bicuspid aortic valve. *Catheter. Cardiovasc. Interv.* 91 986–994. 10.1002/ccd.27467 29399947 PMC5947734

[B82] Lluri G. Renella P. Finn J. P. Vorobiof G. Aboulhosn J. Deb A. (2017). Prognostic significance of left ventricular fibrosis in patients with congenital bicuspid aortic valve. *Am. J. Cardiol.* 120 1176–1179. 10.1016/j.amjcard.2017.06.060 28802508 PMC5593788

[B83] Loeys B. L. Schwarze U. Holm T. Callewaert B. L. Thomas G. H. Pannu H. (2006). Aneurysm syndromes caused by mutations in the TGF-beta receptor. *N. Engl. J. Med.* 355 788–798. 10.1056/NEJMoa055695 16928994

[B84] Longobardo L. Jain R. Carerj S. Zito C. Khandheria B. K. (2016). Bicuspid aortic valve: unlocking the morphogenetic puzzle. *Am. J. Med.* 129 796–805. 10.1016/j.amjmed.2016.03.009 27059385

[B85] MacCarrick G. Black J. R. Bowdin S. El-Hamamsy I. Frischmeyer-Guerrerio P. A. Guerrerio A. L. (2014). Loeys-Dietz syndrome: a primer for diagnosis and management. *Genet. Med.* 16 576–587. 10.1038/gim.2014.11 24577266 PMC4131122

[B86] MacGrogan D. D’Amato G. Travisano S. Martinez-Poveda B. Luxan G. Del M. G. (2016). Sequential ligand-dependent notch signaling activation regulates valve primordium formation and morphogenesis. *Circ. Res.* 118 1480–1497. 10.1161/CIRCRESAHA.115.308077 27056911

[B87] Mart C. R. McNerny B. E. (2013). Shape of the dilated aorta in children with bicuspid aortic valve. *Ann. Pediatr. Cardiol.* 6 126–131. 10.4103/0974-2069.115253 24688228 PMC3957440

[B88] Masri A. Kalahasti V. Alkharabsheh S. Svensson L. G. Sabik J. F. Roselli E. E. (2016). Characteristics and long-term outcomes of contemporary patients with bicuspid aortic valves. *J. Thorac. Cardiovasc. Surg.* 151 1650–1659. 10.1016/j.jtcvs.2015.12.019 26825434

[B89] Masri A. Kalahasti V. Svensson L. G. Alashi A. Schoenhagen P. Roselli E. E. (2017a). Aortic cross-sectional area/height ratio and outcomes in patients with bicuspid aortic valve and a dilated ascending aorta. *Circ. Cardiovasc. Imaging* 10:e6249. 10.1161/CIRCIMAGING.116.006249 28592593

[B90] Masri A. Svensson L. G. Griffin B. P. Desai M. Y. (2017b). Contemporary natural history of bicuspid aortic valve disease: a systematic review. *Heart* 103 1323–1330. 10.1136/heartjnl-2016-309916 28490615

[B91] Mazine A. El-Hamamsy I. Verma S. Peterson M. D. Bonow R. O. Yacoub M. H. (2018). Ross procedure in adults for cardiologists and cardiac surgeons: JACC state-of-the-art review. *J. Am. Coll. Cardiol.* 72 2761–2777. 10.1016/j.jacc.2018.08.2200 30497563

[B92] McMullan D. M. Oppido G. Davies B. Kawahira Y. Cochrane A. D. D’Udekem D. Y. (2007). Surgical strategy for the bicuspid aortic valve: tricuspidization with cusp extension versus pulmonary autograft. *J. Thorac. Cardiovasc. Surg.* 134 90–98. 10.1016/j.jtcvs.2007.01.054 17599491

[B93] Merkx R. Duijnhouwer A. L. Vink E. Roos-Hesselink J. W. Schokking M. (2017). Aortic diameter growth in children with a bicuspid aortic valve. *Am. J. Cardiol.* 120 131–136. 10.1016/j.amjcard.2017.03.245 28483205

[B94] Michelena H. I. Khanna A. D. Mahoney D. Margaryan E. Topilsky Y. Suri R. M. (2011). Incidence of aortic complications in patients with bicuspid aortic valves. *JAMA* 306 1104–1112. 10.1001/jama.2011.1286 21917581

[B95] Michelena H. I. Prakash S. K. Della C. A. Bissell M. M. Anavekar N. Mathieu P. (2014). Bicuspid aortic valve: identifying knowledge gaps and rising to the challenge from the International Bicuspid Aortic Valve Consortium (BAVCon). *Circulation* 129 2691–2704. 10.1161/CIRCULATIONAHA.113.007851 24958752 PMC4145814

[B96] Mojazi-Amiri H. Pai R. G. (2013). Prognostic value of cardiac magnetic resonance imaging in patients with aortic regurgitation. *Future Cardiol.* 9 9–12. 10.2217/fca.12.79 23259471

[B97] Mylotte D. Lefevre T. Sondergaard L. Watanabe Y. Modine T. Dvir D. (2014). Transcatheter aortic valve replacement in bicuspid aortic valve disease. *J. Am. Coll. Cardiol.* 64 2330–2339. 10.1016/j.jacc.2014.09.039 25465419

[B98] Nataatmadja M. West M. West J. Summers K. Walker P. Nagata M. (2003). Abnormal extracellular matrix protein transport associated with increased apoptosis of vascular smooth muscle cells in Marfan syndrome and bicuspid aortic valve thoracic aortic aneurysm. *Circulation* 108(Suppl. 1) I329–I334. 10.1161/01.cir.0000087660.82721.15 12970255

[B99] Navarra E. El K. G. Glineur D. Boodhwani M. Van Dyck M. Vanoverschelde J. L. (2013). Effect of annulus dimension and annuloplasty on bicuspid aortic valve repair. *Eur. J. Cardiothorac. Surg.* 44 316–322; discussion 322–323. 10.1093/ejcts/ezt045 23475588

[B100] Nawaytou O. Mastrobuoni S. de Kerchove L. Baert J. Boodhwani M. El K. G. (2018). Deep circumferential annuloplasty as an adjunct to repair regurgitant bicuspid aortic valves with a dilated annulus. *J. Thorac. Cardiovasc. Surg.* 156 590–597. 10.1016/j.jtcvs.2018.03.110 29887391

[B101] Ng A. C. Delgado V. Bertini M. Antoni M. L. van Bommel R. J. van Rijnsoever E. P. (2011). Alterations in multidirectional myocardial functions in patients with aortic stenosis and preserved ejection fraction: a two-dimensional speckle tracking analysis. *Eur. Heart J.* 32 1542–1550. 10.1093/eurheartj/ehr084 21447510

[B102] Nguyen D. H. Vo A. T. Le K. M. Vu T. T. Nguyen T. T. Vu T. T. (2018). Minimally invasive Ozaki procedure in aortic valve disease: the preliminary results. *Innovations* 13 332–337. 10.1097/IMI.0000000000000556 30394956

[B103] Nishimura R. A. Otto C. M. Bonow R. O. Carabello B. A. Erwin J. R. Fleisher L. A. (2017). 2017 AHA/ACC focused update of the 2014 AHA/ACC guideline for the management of patients with valvular heart disease: a report of the American college of cardiology/American heart association task force on clinical practice guidelines. *J. Am. Coll. Cardiol.* 70 252–289. 10.1016/j.jacc.2017.03.011 28315732

[B104] Nishimura R. A. Otto C. M. Bonow R. O. Carabello B. A. Erwin J. R. Guyton R. A. (2014). 2014 AHA/ACC guideline for the management of patients with valvular heart disease: a report of the American college of cardiology/American heart association task force on practice guidelines. *Circulation* 129 e521–e643. 10.1161/CIR.0000000000000031 24589853

[B105] Nistri S. Porciani M. C. Attanasio M. Abbate R. Gensini G. F. Pepe G. (2012). Association of Marfan syndrome and bicuspid aortic valve: frequency and outcome. *Int. J. Cardiol.* 155 324–325. 10.1016/j.ijcard.2011.12.009 22225761

[B106] Nucifora G. Miller J. Gillebert C. Shah R. Perry R. Raven C. (2018). Ascending aorta and myocardial mechanics in patients with “Clinically Normal” bicuspid aortic valve. *Int. Heart J.* 59 741–749. 10.1536/ihj.17-230 29877299

[B107] Okita Y. (2015). Surgery for thoracic aortic disease in Japan: evolving strategies toward the growing enemies. *Gen. Thorac. Cardiovasc. Surg.* 63 185–196. 10.1007/s11748-014-0476-9 25287705

[B108] Ouzounian M. Mazine A. David T. E. (2017). The Ross procedure is the best operation to treat aortic stenosis in young and middle-aged adults. *J. Thorac. Cardiovasc. Surg.* 154 778–782. 10.1016/j.jtcvs.2017.03.156 28625775

[B109] Ouzounian M. Rao V. Manlhiot C. Abraham N. David C. Feindel C. M. (2016). Valve-sparing root replacement compared with composite valve graft procedures in patients with aortic root dilation. *J. Am. Coll. Cardiol.* 68 1838–1847. 10.1016/j.jacc.2016.07.767 27765186

[B110] Ozaki S. Kawase I. Yamashita H. Uchida S. Nozawa Y. Takatoh M. (2014). A total of 404 cases of aortic valve reconstruction with glutaraldehyde-treated autologous pericardium. *J. Thorac. Cardiovasc. Surg.* 147 301–306. 10.1016/j.jtcvs.2012.11.012 23228404

[B111] Padang R. Bagnall R. D. Richmond D. R. Bannon P. G. Semsarian C. (2012). Rare non-synonymous variations in the transcriptional activation domains of GATA5 in bicuspid aortic valve disease. *J. Mol. Cell. Cardiol.* 53 277–281. 10.1016/j.yjmcc.2012.05.009 22641149

[B112] Padang R. Gersh B. J. Ommen S. R. Geske J. B. (2018). Prevalence and impact of coexistent bicuspid aortic valve in hypertrophic cardiomyopathy. *Heart Lung Circ.* 27 33–40. 10.1016/j.hlc.2017.01.020 28377231

[B113] Paloschi V. Gadin J. R. Khan S. Bjorck H. M. Du L. Maleki S. (2015). Aneurysm development in patients with a bicuspid aortic valve is not associated with transforming growth factor-beta activation. *Arterioscler. Thromb. Vasc. Biol.* 35 973–980. 10.1161/ATVBAHA.114.304996 25745062

[B114] Park J. Y. Foley T. A. Bonnichsen C. R. Maurer M. J. Goergen K. M. Nkomo V. T. (2017). Transthoracic echocardiography versus computed tomography for ascending aortic measurements in patients with bicuspid aortic valve. *J. Am. Soc. Echocardiogr.* 30 625–635. 10.1016/j.echo.2017.03.006 28501375

[B115] Patel P. A. Gutsche J. T. Vernick W. J. Giri J. S. Ghadimi K. Weiss S. J. (2015). The functional aortic annulus in the 3D era: focus on transcatheter aortic valve replacement for the perioperative echocardiographer. *J. Cardiothorac. Vasc. Anesth.* 29 240–245. 10.1053/j.jvca.2014.05.027 25620147

[B116] Perlman G. Y. Blanke P. Dvir D. Pache G. Modine T. Barbanti M. (2016). Bicuspid aortic valve stenosis: favorable early outcomes with a next-generation transcatheter heart valve in a multicenter study. *JACC Cardiovasc. Interv.* 9 817–824. 10.1016/j.jcin.2016.01.002 27101906

[B117] Philip F. Faza N. N. Schoenhagen P. Desai M. Y. Tuzcu E. M. Svensson L. G. (2015). Aortic annulus and root characteristics in severe aortic stenosis due to bicuspid aortic valve and tricuspid aortic valves: implications for transcatheter aortic valve therapies. *Catheter. Cardiovasc. Interv.* 86 E88–E98. 10.1002/ccd.25948 25914355

[B118] Phillippi J. A. Klyachko E. A. Kenny J. T. Eskay M. A. Gorman R. C. Gleason T. G. (2009). Basal and oxidative stress-induced expression of metallothionein is decreased in ascending aortic aneurysms of bicuspid aortic valve patients. *Circulation* 119 2498–2506. 10.1161/CIRCULATIONAHA.108.770776 19398671 PMC5268483

[B119] Pibarot P. Dumesnil J. G. (2012). Improving assessment of aortic stenosis. *J. Am. Coll. Cardiol.* 60 169–180. 10.1016/j.jacc.2011.11.078 22789881

[B120] Poh C. L. Buratto E. Larobina M. Wynne R. O’Keefe M. Goldblatt J. (2018). The Ross procedure in adults presenting with bicuspid aortic valve and pure aortic regurgitation: 85% freedom from reoperation at 20 years. *Eur. J. Cardiothorac. Surg.* 54 420–426. 10.1093/ejcts/ezy073 29546380

[B121] Puranik R. Tsang V. T. Broadley A. Nordmeyer J. Lurz P. Muthialu N. (2010). Functional outcomes after the Ross (pulmonary autograft) procedure assessed with magnetic resonance imaging and cardiopulmonary exercise testing. *Heart* 96 304–308. 10.1136/hrt.2009.172965 19542074

[B122] Reece T. B. Welke K. F. O’Brien S. Grau-Sepulveda M. V. Grover F. L. Gammie J. S. (2014). Rethinking the ross procedure in adults. *Ann. Thorac. Surg.* 97 175–181. 10.1016/j.athoracsur.2013.07.036 24070703

[B123] Renard M. Callewaert B. Malfait F. Campens L. Sharif S. Del C. M. (2013). Thoracic aortic-aneurysm and dissection in association with significant mitral valve disease caused by mutations in TGFB2. *Int. J. Cardiol.* 165 584–587. 10.1016/j.ijcard.2012.09.029 23102774

[B124] Reuthebuch O. Koechlin L. Schurr U. Grapow M. Fassl J. Eckstein F. S. (2018). Aortic valve replacement using autologous pericardium: single centre experience with the Ozaki technique. *Swiss Med. Wkly.* 148:w14591. 10.4414/smw.2018.14591 29442340

[B125] Roman M. J. Pugh N. L. Devereux R. B. Eagle K. A. Holmes K. LeMaire S. A. (2017). Aortic dilatation associated with bicuspid aortic valve: relation to sex, hemodynamics, and valve morphology (the National Heart Lung and Blood Institute-Sponsored National Registry of Genetically Triggered Thoracic Aortic Aneurysms and Cardiovascular Conditions). *Am. J. Cardiol.* 120 1171–1175. 10.1016/j.amjcard.2017.06.061 28802510 PMC5593782

[B126] Ruzmetov M. Shah J. J. Fortuna R. S. Welke K. F. (2015). The association between aortic valve leaflet morphology and patterns of aortic dilation in patients with bicuspid aortic valves. *Ann. Thorac. Surg.* 99 2101–2107; discussion 2107–2108. 10.1016/j.athoracsur.2015.02.036 25921253

[B127] Sannino A. Cedars A. Stoler R. C. Szerlip M. Mack M. J. Grayburn P. A. (2017). Comparison of efficacy and safety of transcatheter aortic valve implantation in patients with bicuspid versus tricuspid aortic valves. *Am. J. Cardiol.* 120 1601–1606. 10.1016/j.amjcard.2017.07.053 28886853

[B128] Schneider U. Hofmann C. Aicher D. Takahashi H. Miura Y. Schafers H. J. (2017). Suture annuloplasty significantly improves the durability of bicuspid aortic valve repair. *Ann. Thorac. Surg.* 103 504–510. 10.1016/j.athoracsur.2016.06.072 27663792

[B129] Shen M. Tastet L. Capoulade R. Larose E. Bedard E. Arsenault M. (2017). Effect of age and aortic valve anatomy on calcification and haemodynamic severity of aortic stenosis. *Heart* 103 32–39. 10.1136/heartjnl-2016-309665 27504001

[B130] Siddiqui J. Brizard C. P. Konstantinov I. E. Galati J. Wheaton G. Cheung M. (2013). Outcomes after operations for bicuspid aortic valve disease in the pediatric population. *Ann. Thorac. Surg.* 96 2175–2183. 10.1016/j.athoracsur.2013.07.130 24182911

[B131] Sievers H. H. Schmidtke C. (2007). A classification system for the bicuspid aortic valve from 304 surgical specimens. *J. Thorac. Cardiovasc. Surg.* 133 1226–1233. 10.1016/j.jtcvs.2007.01.039 17467434

[B132] Sievers H. H. Stierle U. Charitos E. I. Takkenberg J. J. Horer J. Lange R. (2016). A multicentre evaluation of the autograft procedure for young patients undergoing aortic valve replacement: update on the German Ross Registrydagger. *Eur. J. Cardiothorac. Surg.* 49 212–218. 10.1093/ejcts/ezv001 25666469

[B133] Sievers H. H. Stierle U. Mohamed S. A. Hanke T. Richardt D. Schmidtke C. (2014). Toward individualized management of the ascending aorta in bicuspid aortic valve surgery: the role of valve phenotype in 1362 patients. *J. Thorac. Cardiovasc. Surg.* 148 2072–2080. 10.1016/j.jtcvs.2014.04.007 24841446

[B134] Siu S. C. Silversides C. K. (2010). Bicuspid aortic valve disease. *J. Am. Coll. Cardiol.* 55 2789–2800. 10.1016/j.jacc.2009.12.068 20579534

[B135] Sotelo J. Dux-Santoy L. Guala A. Rodriguez-Palomares J. Evangelista A. Sing-Long C. (2018). 3D axial and circumferential wall shear stress from 4D flow MRI data using a finite element method and a laplacian approach. *Magn. Reson. Med.* 79 2816–2823. 10.1002/mrm.26927 28980342

[B136] Spaziani G. Ballo P. Favilli S. Fibbi V. Buonincontri L. Pollini I. (2014). Clinical outcome, valve dysfunction, and progressive aortic dilation in a pediatric population with isolated bicuspid aortic valve. *Pediatr. Cardiol.* 35 803–809. 10.1007/s00246-013-0856-4 24362596

[B137] Sun B. J. Lee S. Jang J. Y. Kwon O. Bae J. S. Lee J. H. (2017). Performance of a simplified dichotomous phenotypic classification of bicuspid aortic valve to predict type of valvulopathy and combined aortopathy. *J. Am. Soc. Echocardiogr.* 30 1152–1161. 10.1016/j.echo.2017.08.002 29066082

[B138] Svensson L. G. Al K. A. Vivacqua A. Pettersson G. B. Gillinov A. M. Mihaljevic T. (2014). Long-term durability of bicuspid aortic valve repair. *Ann. Thorac. Surg.* 97 1539–1547; discussion 1548. 10.1016/j.athoracsur.2013.11.036 24680032

[B139] Tang P. C. Badami A. Akhter S. A. Osaki S. Lozonschi L. Kohmoto T. (2017). Efficacy of aortic valve resuspension in establishing valve competence in acute type a dissections. *Ann. Thorac. Surg.* 103 1460–1466. 10.1016/j.athoracsur.2016.08.099 27863732

[B140] Thaden J. J. Nkomo V. T. Lee K. J. Oh J. K. (2015). Doppler imaging in aortic stenosis: the importance of the nonapical imaging windows to determine severity in a contemporary cohort. *J. Am. Soc. Echocardiogr.* 28 780–785. 10.1016/j.echo.2015.02.016 25857547

[B141] Theodoris C. V. Li M. White M. P. Liu L. He D. Pollard K. S. (2015). Human disease modeling reveals integrated transcriptional and epigenetic mechanisms of NOTCH1 haploinsufficiency. *Cell* 160 1072–1086. 10.1016/j.cell.2015.02.035 25768904 PMC4359747

[B142] Treibel T. A. Lopez B. Gonzalez A. Menacho K. Schofield R. S. Ravassa S. (2018). Reappraising myocardial fibrosis in severe aortic stenosis: an invasive and non-invasive study in 133 patients. *Eur. Heart J.* 39 699–709. 10.1093/eurheartj/ehx353 29020257 PMC5888951

[B143] Van Hemelrijk C. Renard M. Loeys B. (2010). The Loeys-Dietz syndrome: an update for the clinician. *Curr. Opin. Cardiol.* 25 546–551. 10.1097/HCO.0b013e32833f0220 20838339

[B144] Verma S. Siu S. C. (2014). Aortic dilatation in patients with bicuspid aortic valve. *N. Engl. J. Med.* 370 1920–1929. 10.1056/NEJMra1207059 24827036

[B145] von Knobelsdorff-Brenkenhoff F. Karunaharamoorthy A. Trauzeddel R. F. Barker A. J. Blaszczyk E. Markl M. (2016). Evaluation of aortic blood flow and wall shear stress in aortic stenosis and its association with left ventricular remodeling. *Circ. Cardiovasc. Imaging* 9:e4038. 10.1161/CIRCIMAGING.115.004038 26917824 PMC4772425

[B146] Wang Y. Wang M. Song G. Wang W. Lv B. Wang H. (2018). Optimal pre-TAVR annulus sizing in patients with bicuspid aortic valve: area-derived perimeter by CT is the best-correlated measure with intraoperative sizing. *Eur. Radiol.* 10.1007/s00330-018-5592-y [Epub ahead of print]. 29926206

[B147] Wang Y. B. Li Y. Deng Y. B. Liu Y. N. Zhang J. Sun J. (2018). Enlarged size and impaired elastic properties of the ascending aorta are associated with endothelial dysfunction and elevated plasma matrix metalloproteinase-2 level in patients with bicuspid aortic valve. *Ultrasound Med. Biol.* 44 955–962. 10.1016/j.ultrasmedbio.2018.01.012 29472114

[B148] Wang Y. Wu B. Dong L. Wang C. Wang X. Shu X. (2016). Circulating matrix metalloproteinase patterns in association with aortic dilatation in bicuspid aortic valve patients with isolated severe aortic stenosis. *Heart Vessels* 31 189–197. 10.1007/s00380-014-0593-5 25325992

[B149] Ward R. M. Marsh J. M. Gossett J. M. Rettiganti M. R. Collins R. N. (2018). Impact of bicuspid aortic valve morphology on aortic valve disease and aortic dilation in pediatric patients. *Pediatr. Cardiol.* 39 509–517. 10.1007/s00246-017-1781-8 29188316

[B150] Weismann C. G. Lombardi K. C. Grell B. S. Northrup V. Sugeng L. (2016). Aortic stiffness and left ventricular diastolic function in children with well-functioning bicuspid aortic valves. *Eur. Heart J. Cardiovasc. Imaging* 17 225–230. 10.1093/ehjci/jev151 26072912 PMC4882883

[B151] Wojnarski C. M. Svensson L. G. Roselli E. E. Idrees J. J. Lowry A. M. Ehrlinger J. (2015). Aortic dissection in patients with bicuspid aortic valve-associated aneurysms. *Ann. Thorac. Surg.* 100 1666–1673; discussion 1673–1674. 10.1016/j.athoracsur.2015.04.126 26209494 PMC5166604

[B152] Wu J. Song H. F. Li S. H. Guo J. Tsang K. Tumiati L. (2016). Progressive aortic dilation is regulated by miR-17-associated miRNAs. *J. Am. Coll. Cardiol.* 67 2965–2977. 10.1016/j.jacc.2016.04.027 27339495

[B153] Xu Y. N. Xiong T. Y. Li Y. J. Liao Y. B. Zhao Z. G. Wei X. (2018). Balloon sizing during transcatheter aortic valve implantation: comparison of different valve morphologies. *Herz* 10.1007/s00059-018-4714-2 [Epub ahead of print]. 29869699

[B154] Xuan Y. Wang Z. Liu R. Haraldsson H. Hope M. D. Saloner D. A. (2018). Wall stress on ascending thoracic aortic aneurysms with bicuspid compared with tricuspid aortic valve. *J. Thorac. Cardiovasc. Surg.* 156 492–500. 10.1016/j.jtcvs.2018.03.004 29656820 PMC8447844

[B155] Yakar T. S. Tuluce K. Simsek E. C. Safak O. Okten M. S. Yapan E. Z. (2017). Assessment of bicuspid aortic valve phenotypes and associated pathologies: a transesophageal echocardiographic study. *Turk Kardiyol. Dern. Ars.* 45 690–701. 10.5543/tkda.2017.03152 29226889

[B156] Yamashita T. Hayashi T. Tabata T. Hirata K. I. (2018). Bicuspid aortic valve-associated aortic dilatation- what is the mechanism of bicuspid aortopathy? *Circ. J.* 82 2470–2471. 10.1253/circj.CJ-18-0844 30101811

[B157] Yang B. Zhou W. Jiao J. Nielsen J. B. Mathis M. R. Heydarpour M. (2017). Protein-altering and regulatory genetic variants near GATA4 implicated in bicuspid aortic valve. *Nat. Commun.* 8:15481. 10.1038/ncomms15481 28541271 PMC5458508

[B158] Yang T. H. Webb J. G. Blanke P. Dvir D. Hansson N. C. Norgaard B. L. (2015). Incidence and severity of paravalvular aortic regurgitation with multidetector computed tomography nominal area oversizing or undersizing after transcatheter heart valve replacement with the Sapien 3: a comparison with the Sapien XT. *JACC Cardiovasc. Interv.* 8 462–471. 10.1016/j.jcin.2014.10.014 25790764

[B159] Yassine N. M. Shahram J. T. Body S. C. (2017). Pathogenic mechanisms of bicuspid aortic valve aortopathy. *Front. Physiol.* 8:687 10.3389/fphys.2017.00687 PMC5622294 28993736

[B160] Yoon S. H. Bleiziffer S. De Backer O. Delgado V. Arai T. Ziegelmueller J. (2017). Outcomes in transcatheter aortic valve replacement for bicuspid versus tricuspid aortic valve stenosis. *J. Am. Coll. Cardiol.* 69 2579–2589. 10.1016/j.jacc.2017.03.017 28330793

[B161] Yoon S. H. Lefevre T. Ahn J. M. Perlman G. Y. Dvir D. Latib A. (2016). Transcatheter aortic valve replacement with early- and new-generation devices in bicuspid aortic valve stenosis. *J. Am. Coll. Cardiol.* 68 1195–1205. 10.1016/j.jacc.2016.06.041 27609682

[B162] Youssefi P. Sharma R. Figueroa C. A. Jahangiri M. (2017). Functional assessment of thoracic aortic aneurysms - the future of risk prediction? *Br. Med. Bull.* 121 61–71. 10.1093/bmb/ldw049 27989994 PMC5862296

[B163] Zacek P. Holubec T. Vobornik M. Dominik J. Takkenberg J. Harrer J. (2016). Quality of life after aortic valve repair is similar to Ross patients and superior to mechanical valve replacement: a cross-sectional study. *BMC Cardiovasc. Disord.* 16:63. 10.1186/s12872-016-0236-0 27039180 PMC4818911

[B164] Zeeshan A. Idrees J. J. Johnston D. R. Rajeswaran J. Roselli E. E. Soltesz E. G. (2018). Durability of aortic valve cusp repair with and without annular support. *Ann. Thorac. Surg.* 105 739–748. 10.1016/j.athoracsur.2017.09.019 29273323

[B165] Zegdi R. Ciobotaru V. Noghin M. Sleilaty G. Lafont A. Latremouille C. (2008). Is it reasonable to treat all calcified stenotic aortic valves with a valved stent? Results from a human anatomic study in adults. *J. Am. Coll. Cardiol.* 51 579–584. 10.1016/j.jacc.2007.10.023 18237689

[B166] Zegri-Reiriz I. de Alarcon A. Munoz P. Martinez S. M. Gonzalez-Ramallo V. Miro J. M. (2018). Infective endocarditis in patients with bicuspid aortic valve or mitral valve prolapse. *J. Am. Coll. Cardiol.* 71 2731–2740. 10.1016/j.jacc.2018.03.534 29903346

[B167] Zhao Z. G. Jilaihawi H. Feng Y. Chen M. (2015). Transcatheter aortic valve implantation in bicuspid anatomy. *Nat. Rev. Cardiol.* 12 123–128. 10.1038/nrcardio.2014.161 25311233

